# A hidden Markov model for lymphatic tumor progression in the head and neck

**DOI:** 10.1038/s41598-021-91544-1

**Published:** 2021-06-10

**Authors:** Roman Ludwig, Bertrand Pouymayou, Panagiotis Balermpas, Jan Unkelbach

**Affiliations:** Department of Radiation Oncology, University Hospital of Zurich, Zurich, Switzerland

**Keywords:** Cancer models, Cancer therapy, Head and neck cancer, Metastasis, Information theory and computation, Computational science, Statistics, Software

## Abstract

Currently, elective clinical target volume (CTV-N) definition for head and neck squamous cell carcinoma (HNSCC) is mostly based on the prevalence of nodal involvement for a given tumor location. In this work, we propose a probabilistic model for lymphatic metastatic spread that can quantify the risk of microscopic involvement in lymph node levels (LNL) given the location of macroscopic metastases and T-category. This may allow for further personalized CTV-N definition based on an individual patient’s state of disease. We model the patient's state of metastatic lymphatic progression as a collection of hidden binary random variables that indicate the involvement of LNLs. In addition, each LNL is associated with observed binary random variables that indicate whether macroscopic metastases are detected. A hidden Markov model (HMM) is used to compute the probabilities of transitions between states over time. The underlying graph of the HMM represents the anatomy of the lymphatic drainage system. Learning of the transition probabilities is done via Markov chain Monte Carlo sampling and is based on a dataset of HNSCC patients in whom involvement of individual LNLs was reported. The model is demonstrated for ipsilateral metastatic spread in oropharyngeal HNSCC patients. We demonstrate the model's capability to quantify the risk of microscopic involvement in levels III and IV, depending on whether macroscopic metastases are observed in the upstream levels II and III, and depending on T-category. In conclusion, the statistical model of lymphatic progression may inform future, more personalized, guidelines on which LNL to include in the elective CTV. However, larger multi-institutional datasets for model parameter learning are required for that.

## Introduction

In radiation therapy and surgical treatment of most cancer types it is the aim to irradiate or resect as much malign tissue as possible, including elective treatment of regions of possible microscopic spread, to increase the patient’s probability of cure^1,2^. Many cancer types spread though the lymphatic system and metastasize in regional lymph nodes^3–8^. Sufficiently large metastases can be identified using computed tomography (CT), magnetic resonance imaging (MRI), or positron emission tomography (PET)^9–11^. However, current in-vivo imaging techniques are not able to detect microscopic metastases, which would require pathological examination of the tissue^12,13^. Clinicians are therefore regularly challenged with assessing the risk of microscopic involvement in regions that are not clearly cancerous. Deciding which part of the lymph drainage region to irradiate or resect is essential to balance the conflicting goals of maximizing the tumor control probability (TCP), while at the same time minimizing harmful side effects associated with unnecessary treatment of healthy tissue^14^.

In this work, we consider head and neck squamous cell carcinomas (HNSCC), which frequently spread through the lymphatic system in the neck region. In case of pharyngeal tumors (hypo-, naso- and oropharynx), between 64 and 80% of patients present with clinical metastatic neck nodes at the time of diagnoses^3,15^. To standardize the location of lymph node metastases, the neck is anatomically divided into lymph node levels (LNL)^16,17^. LNLs are then often prophylactically irradiated or resected based on the possibility of harboring occult metastases despite negative findings on imaging. In the case of radiotherapy, this concept is referred to as elective nodal irradiation. Defining the nodal clinical target volume (CTV-N) for radiotherapy planning amounts to deciding which LNLs to include in the CTV-N. Current guidelines^18–25^ are mostly based on reports^6,7,23,26–30^ regarding the prevalence of lymph node involvement in these levels for a given location of the primary tumor, and thus on the patterns of lymphatic progression that were previously observed.

However, current guidelines do not provide clinicians with personalized risk assessments on an individual patient basis. Prevalence of lymph node involvement in a population of patients does not quantify the risk of microscopic involvement for any particular patient presenting with a specific state of tumor progression. For example, a patient presenting with macroscopic lymph node metastases visible in PET-CT in levels II and III may have a higher risk of harboring occult metastases in level IV compared to a patient without diagnosed metastases in level III. To address this aspect, a methodology for quantitative risk assessment has been proposed that uses Bayesian networks to model the joint probability distribution of LNL involvement^31^ based on a dataset of lymphatic progression pattern in a cohort of HNSCC patients^8^.

However, the mentioned work on a Bayesian network model^31^ was not able to describe the evolution of a patient’s disease over time in a natural manner. Our work can be seen as an extension of this earlier work^31^ in that respect and extends its capabilities regarding the incorporation of T-category into the risk estimation of microscopic involvement. Metastatic progression of tumors is a dynamic process in which the probability of LNL involvement increases over time. We introduce a probabilistic model of lymphatic metastatic spread over time using hidden Markov models (HMM). The T-category of a tumor can be seen as a surrogate for time. Early and late T-category tumors are the same type of tumors with the same patterns of lymphatic progression, with the main difference that tumors with advanced T-category are on average diagnosed at a later point in time. The model is trained with detailed involvement patterns from a cohort of HNSCC patients and can afterwards be used to predict the risk of nodal involvement for new patients, given their T-category and location of macroscopic metastases.

In "Bayesian network of lymph node level involvement" section we introduce notation and briefly recap how Bayesian networks (BN) were used previously^31^ to model lymphatic spread, which is the foundation for the further development presented in this paper. Afterwards in "Hidden Markov model of lymphatic tumor progression" section, we describe in detail the mathematics of how we applied hidden Markov models (HMM) to model tumor progression over time and incorporate T-category into microscopic involvement risk estimation. How we tested our model’s predictive capabilities is described in "Application to oropharyngeal HNSCC" section along with the respective results. Finally—in "Discussion and Outlook" section—we will discuss future steps towards improving the methodology further. We also make the code base that was developed and used for this work publicly available along with the data the model was trained on for the results presented here (see supplementary information).

## Bayesian network of lymph node level involvement

We model the state of each LNL as a hidden or unobserved binary random variable, which indicates via values 0 or 1 if an LNL is healthy or involved, respectively. This state indicates if there is truly tumor present in an LNL, including the presence of occult metastases for the involved state—motivating the term hidden or unobserved state. Every LNL can be diagnosed using one or multiple modalities. Most used for diagnosis are imaging techniques like PET, CT and MRI, but palpation or fine needle aspiration (FNA) are also used. The diagnosis too, is modelled as binary random variable—this time an observed one—taking on 0 for *negative* and 1 for *positive*.

For notational convenience, we collect the hidden and observed random variables in a random vector each:

$$\begin{aligned}
\text{hidden}& \qquad \boldsymbol{X} = (X_v) \rightarrow \{0,1\}^V \\
\text{observed}& \qquad \boldsymbol{Z} = (Z_v^k) \rightarrow \{0,1\}^{V \times |\mathcal{O}|} 
\end{aligned}$$

where $V$ is the number of LNLs $v \in \left\{ {1,2, \ldots ,V} \right\}$ in the graph, while we have called the set of diagnostic modalities ${\mathcal{O}} = \left\{ {{\text{CT}},{\text{MRI}},{\text{palpation}},{\text{FNA}}, \ldots } \right\}$.

The conditional probabilities that link the hidden state to the observations can be written as follows:

1 $$\begin{aligned}
P_{BN}\left( Z_v^k = z_v^k \mid X_v = x_v \right) =& \left( z_v^k + (-1)^{z_v^k} \cdot s_P^k \right) (1 - x_v) \\
+& \left( (1 - z_v^k) + (-1)^{1 - z_v^k} \cdot s_N^k \right) x_v
\end{aligned}$$

with $s_{N}^{k}$ and $s_{P}^{k}$ being the sensitivity and specificity of the diagnostic method, respectively. For example, for the probability of a false negative observation, that is diagnostic modality $k$ misses the presence of tumor, we get

2 $$P_{BN} \left( Z_v^k = 0 \mid X_v = 1 \right) = 1 - s_N^k$$

**Figure 1:**
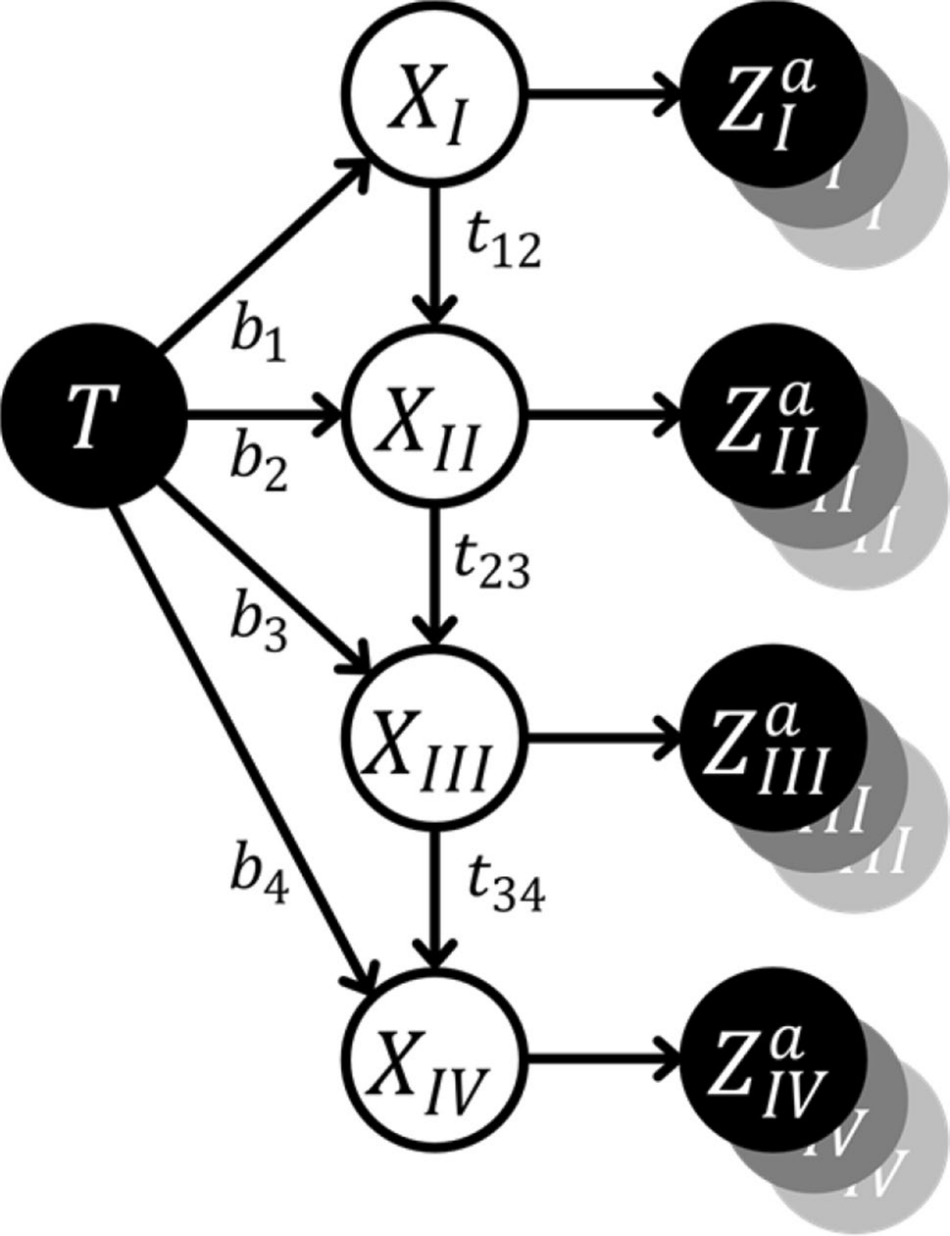
Bayesian network for modelling lymphatic metastatic spread as described by (Pouymayou et al.)^31^. It consists of the primary tumor $T$, hidden binary variables $X_{v}$ for the involvement of LNL $v$ (white circles) and observed (or diagnostic) variables as dark circles ($Z_{v}^{{\mathcal{O}}}$, where ${\mathcal{O}}$ denotes the used diagnostic modality). There are potentially many observations per hidden variable. Annotated arcs depict the direction of lymphatic flow where the parameter next to it ($b$ and $t$) represents the probability of metastatic spread. Not annotated arrows connect the LNLs to their diagnoses via sensitivity and specificity.

Spread of the tumor through the lymphatic network is represented in this model by directed arcs to and between LNLs as illustrated in Fig. 1. We introduce an additional vertex to the graph representing the primary tumor, which we assume to be the only one. Directed arcs from the primary tumor to an LNL represent direct spread of tumor cells from the primary tumor to the LNL. These arcs are associated with parameters $b_{v}$ that we call *base probabilities,* and which indicate the probability that the tumor spreads directly to LNL $v$. When LNL $s$ receives efferent lymphatics from LNL $r$, this too is represented by a directed arc from LNL $r$ to $s$, and $r = {\text{pa}}\left( s \right)$ which is called a parent node of $s$. These arcs are associated with a *transition probability* $t_{rs}$ from $r$ to $s$. The network shown in Fig. 1, comprising ipsilateral levels I, II, II, and IV, will be used throughout this work. However, when more data of detailed LNL involvement including additional levels becomes available and/or contralateral involvement, the model can be extended.

The parameters $b_{v}$ and $t_{rs}$ associated with the directed arcs represent conditional probabilities, i.e. $b_{v}$ answers the question *given that all parent nodes are healthy, how likely is it that the primary tumor spreads to node* $v$*?* $t_{rs}$ on the other hand, can answer the question *assuming no efferent spread from the primary tumor and given that all parent nodes except* $r$ *are healthy, what is the likelihood of spread to node* $s$*?* The conditional probability for involvement of LNL $v$ given the state of its parent nodes is then given by

3 $$P_BN \left( X_V = x_v \mid X_{\operatorname{pa}(v)} = x_{\operatorname{pa}(v)}, b_v, t_{\operatorname{pa}(v)v} \right) = x_v + (-1)^{x_v} (1 - b_v) (1 - t_{\operatorname{pa}(v)v})^{x_{\operatorname{pa}(v)}}$$

We note here that this parametrization assumes the independence of causal influences (ICI), thereby allowing us to describe the model using only a few interpretable parameters. Dropping this assumption, a BN can also be defined using conditional probability tables (CPT) that have columns for every possible combinations of parent states^32^. However, with the increase of the number of parent nodes (causes) in the graph, the number of parameters in the respective CPT would grow exponentially.

For the graph in Fig. 1 we can write down the parametrized CPT in the following manner:

4 $$\begin{aligned}
P_{BN} \left( X_v = 0 \mid X_{\operatorname{pa}(v)} = 0 \right) &= 1 - b_v \\
P_{BN} \left( X_v = 1 \mid X_{\operatorname{pa}(v)} = 0 \right) &= b_v \\
P_{BN} \left( X_v = 0 \mid X_{\operatorname{pa}(v)} = 1 \right) &= (1 - b_v) (1 - t_{\operatorname{pa}(v)v}) \\
P_{BN} \left( X_v = 1 \mid X_{\operatorname{pa}(v)} = 1 \right) &= 1 - (1 - b_v) (1 - t_{\operatorname{pa}(v)v}) \\
\end{aligned}$$

In case of a more general network, in which some LNLs receive efferent lymphatics from multiple other LNLs, Eq. (3) can be generalized and the conditional probability of the hidden state becomes

5 $$P_{BN} \left( X_v = x_v \mid \left\{ X_{\operatorname{pa}(v)} = x_{\operatorname{pa}(v)} \right\}, \left\{ t_{\operatorname{pa}(v)v} \right\}, b_v \right) = x_v + (-1)^{x_v} (1 - b_v) \prod_{p \in \operatorname{pa}(v)}{ (1 - t_{pv})^{x_p} }$$

We can now connect the probability of observing certain $Z_{v}^{k}$ given the hidden involvement with the conditional probabilities above. Then the likelihood of observing a cohort of patients ${\mathbf{\mathcal{Z}}} = \left\{ {z_{nv}^{k} |n \le N,v \le V,k \in {\mathcal{O}}} \right\}$ given a set of parameters $\theta = \left\{ {b_{v} , t_{pa\left( v \right)v} |v \le V} \right\}$ is given by

6 $$P_{BN} \left( \mathcal{Z} \mid \theta \right) = \prod_{n=1}^{N}{ \sum_{\boldsymbol{x} \in \left\{ 0,1 \right\}^V}{ \prod_{v=1}^V{ \prod_{k \in \mathcal{O}}{ P_{BN} \left( z_{nv}^k \mid x_v \right) P_{BN} \left( x_v \mid \left\{ x_{\operatorname{pa}(v)} \right\}, \left\{ t_{\operatorname{pa}(v)v} \right\}, b_v \right)} } } }$$

where we marginalized over all hidden variables $X$. Here we have assumed that each patient’s diagnosis ${\varvec{z}} = \left\{ {z_{v}^{k} \, | \, v \le V,k \in {\mathcal{O}}} \right\}$ is complete, meaning that for all possible observation/imaging modalities, we have a diagnosis for each LNL. The likelihood can then be used to infer the model parameters via maximum likelihood inference or sampling.

## Hidden Markov model of lymphatic tumor progression

While Bayesian networks can model the probabilistic relationship between involvement in different levels, they lack an explicit way to describe the evolution of the tumor over time. The concept of *dynamic Bayesian network* (DBN) has been developed to introduce the notion of time into probabilistic models. DBNs are generalizations of hidden Markov models^33^ and formally similar to what we will introduce now. The metastatic spread in the lymphatic system is a dynamic system and by modelling it with a formalism that can capture this, we obtain a more intuitive model of the problem and a framework that can incorporate T-category into estimating the risk of LNL involvement. We can do this because tumors go through the stages T1 to T4 sequentially, meaning that—for a given tumor—it is a surrogate of time.

### Formulating lymphatic progression as HMM

**Figure 2:**
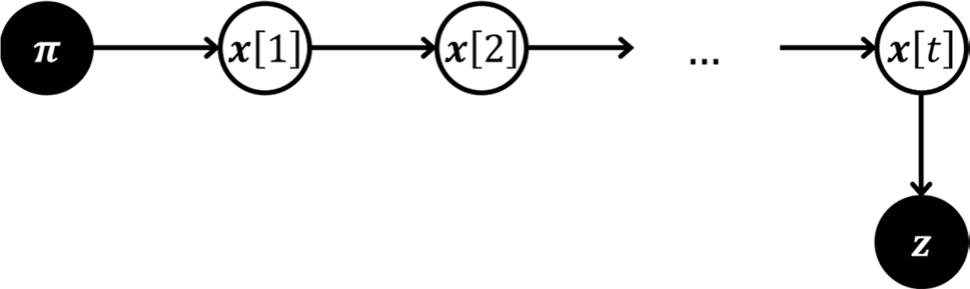
Hidden Markov model with only one observation. ${\varvec{\pi}}$ denotes the healthy starting state. Horizontal arcs represent the transitions from a state. (${\varvec{x}}\left[ {t - 1} \right]$) to the state at the next time step (${\varvec{x}}\left[ t \right]$). The final state is then diagnosed (vertical arc, parametrized via sensitivity and specificity) and we observe ${\varvec{z}}$.

We consider discrete time-steps $t \in \left\{ {0, 1, 2, \ldots , T} \right\}$. We will start by defining the hidden random variable for the state of the HMM at time $t$ to be

7 $$\begin{array}{*{20}c} {{\varvec{X}}\left[ t \right] = \left( {X_{v} \left[ t \right]} \right)} \\ \end{array}$$

which represents the patient’s state of LNL involvement as in the BN, but for each time-step we have an instance of it. For the diagnosis ${\varvec{Z}}$ on the other hand, we do not need to differentiate between different times, since in practice we will only ever see one diagnosis. This is illustrated in Fig. 2. The reason for this is that, if we diagnose a patient with cancer, treatment starts timely and we no longer observe the natural progression of the disease. From a modelling standpoint however, this is a problem that we will address later.

A hidden Markov model is fully described by the starting state ${\varvec{X}}\left[ 0 \right]: = {\varvec{\pi}}$ and the two conditional probability functions that govern the progression from a state $X\left[ t \right]$ at time $t$ to a state $X\left[ {t + 1} \right]$ at the following time-step

8 $$\begin{array}{*{20}c} {P_{HMM} \left( {{\varvec{X}}\left[ {t + 1} \right] \, | \, {\varvec{X}}\left[ t \right]} \right)} \\ \end{array}$$

and the probability of a diagnostic observation given the true state of the patient

9 $$\begin{array}{*{20}c} {P_{HMM} \left( {{\varvec{Z}} \, | \, {\varvec{X}}\left[ t \right]} \right)} \\ \end{array}$$

Since both our state space and our observation space are discrete and finite, it is possible to enumerate all possible states and observations and collect them in a table or matrix. The *transition matrix* would then be

10 $$\begin{array}{*{20}c} {A = \left( {a_{ij} } \right) = \left( {P_{HMM} \left( {{\varvec{X}}\left[ {t + 1} \right] = {\varvec{\xi}}_{i} \, | \, {\varvec{X}}\left[ t \right] = {\varvec{\xi}}_{j} } \right)} \right)} \\ \end{array}$$

and the *observation matrix*

11 $$\begin{array}{*{20}c} {B = \left( {b_{ij} } \right) = \left( {P_{HMM} \left( {{\varvec{Z}} = {\varvec{\zeta}}_{j} \user2{\, | \, X}\left[ t \right] = {\varvec{\xi}}_{i} } \right)} \right)} \\ \end{array}$$

**Figure 3:**
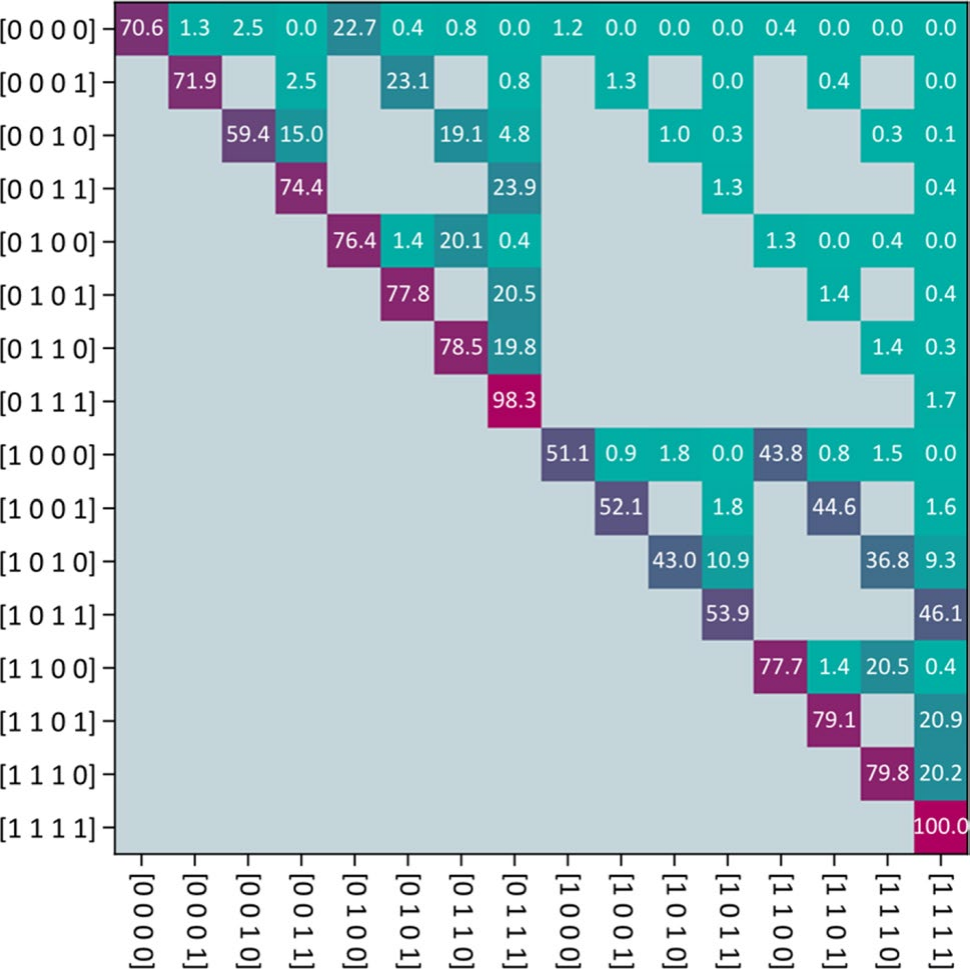
Transition matrix. All gray pixels in this image correspond to entries in the matrix being zero. The colored pixels take on values $\in \left[ {0,1} \right]$ which are here overlayed in %. The exact values stem from the mean of the learned parameters in "Application to oropharyngeal HNSCC" section. The exact shape of the grey “mask” depends on how one orders the states.

Here ${\varvec{\xi}}_{i}$ and ${\varvec{\zeta}}_{j}$ are no new variables, just ${\varvec{x}}$ and ${\varvec{z}}$ renamed and reordered. The indices $i$ and $j$ are for one of the possible states or observations for the entire patient, not for an individual LNL. In total, there are $S = \left| {\left\{ {0,1} \right\}} \right|^{V}$ different states and $S^{{\left| {\mathcal{O}} \right|}} = \left| {\left\{ {0,1} \right\}} \right|^{{V \cdot \left| {\mathcal{O}} \right|}}$ different possible observations. We order the hidden states from

12 $$\begin{array}{*{20}c} {{\varvec{\xi}}_{1} = \left( {\begin{array}{*{20}c} 0 & 0 & 0 & 0 \\ \end{array} } \right)} \\ \end{array}$$

to

13 $$\begin{array}{*{20}c} {{\varvec{\xi}}_{16} = \left( {\begin{array}{*{20}c} 1 & 1 & 1 & 1 \\ \end{array} } \right)} \\ \end{array}$$

in the case of $V = 4$. The exact ordering does not matter, it is just a convenience for the notation. Our ordering of the states can be seen in the axes of Fig. 3. In analogy, we order the observations ${\varvec{\zeta}}_{j}$ from $1$ to $V \cdot \left| {\mathcal{O}} \right|$.

In our case, the starting state corresponds to a primary tumor being present but all LNLs are still in the healthy state. The observation matrix ${\varvec{B}}$ is specified via sensitivity and specificity as described in Eq. (11). The main task is to infer the transition matrix ${\varvec{A}}$. Usually, it is inferred from a series of observations and there exist efficient algorithms for that, e.g. the *sum-product algorithm,* which is particularly efficient in chains. Unfortunately, these algorithms cannot be applied for our problem for two profound reasons:

- We only have a single observation instead of a consecutive series of observations.
- It is unclear how many time-steps it took from the starting state to the one observation we have at the time of diagnosis.

In the remainder of "Hidden Markov model of lymphatic tumor progression" section, we will detail the HMM step-by-step, starting with the parameterization of the transition matrix ${\varvec{A}}$ in "Parametrization of the transition matrix" section. Afterwards, "Marginalization" section will tackle problems (a) and (b), followed up by explaining how we perform inference on this model ("Inference of model parameters" section), incorporate information about a patient’s T-category ("Incorporation of T-category" section) and assess the risk of LNL involvement in a new patient ("Risk assessment of microscopic involvement" section). Lastly, we will introduce a way to incorporate incomplete observations in "Learning and risk assessment for incomplete diagnoses" section.

### Parametrization of the transition matrix

The transition matrix ${\varvec{A}}$ has $S = 2^{2V}$ entries and therefore $S\left( {S - 1} \right) = 2^{2V} - 2^{V}$ degrees of freedom. Although searching the full space of viable transition matrices is possible via unparametrized sampling techniques, it is computationally challenging and hard to interpret. To achieve this reduction in degrees of freedom, and also preserve the anatomically and medically motivated structure of the Bayesian network in "Bayesian network of lymph node level involvement" section, we can represent the transition probability from one state ${\varvec{x}}\left[ t \right]$ to another state ${\varvec{x}}\left[ {t + 1} \right]$ using the conditional probabilities defined for the BN. The difference is that the probability of observing a certain state of LNL $v$ now depends on the state of the patient one time-step before. Note that from here on, we will mostly drop the probabilistically correct notation $P\left( {X = x} \right)$ and just write $P\left( x \right)$ for brevity.

14 $$P_{HMM} \left( \boldsymbol{x}[t + 1] \mid \boldsymbol{x}[t] \right) = \prod_{v \in V}{ Q\left( x_v[t + 1]; x_v[t] \right) \left( P_{BN} \left( x_v[t+1] \mid \left\{ x_{\operatorname{pa}(v)}[t] \right\}, \left\{ \tilde{t}_{\operatorname{pa}(v)v} \right\}, \tilde{b}_v \right) \right)^{1 - x_v[t]} }$$

Here we have reused the conditional probability from the Bayesian network for each LNL, but we take it to the power of one minus that node’s previous value. This ensures that an involved node stays involved with probability 1. The parameters $\tilde{t}_{{{\text{pa}}\left( v \right)v}}$ and $\tilde{b}_{v}$ take the same role as in the BN, but they are now probability *rates*, since they act per time-step. Lastly, the first term $Q$ in the product formalizes the fact that a metastatic lymph node level cannot become healthy again once it was involved. This also means that several entries in the transition matrix ${\varvec{A}}$ must be zero. In a table the values of $Q\left( {x_{v} \left[ {t + 1} \right];x_{v} \left[ t \right]} \right)$ can be written like this:

15 $$\begin{aligned}
Q \left( X_v[t + 1] = 0; X_v[t] = 0 \right) &= 1 \\
Q \left( X_v[t + 1] = 0; X_v[t] = 1 \right) &= 0 \\
Q \left( X_v[t + 1] = 1; X_v[t] = 0 \right) &= 1 \\
Q \left( X_v[t + 1] = 1; X_v[t] = 1 \right) &= 1
\end{aligned}$$

which gives effectively rise to a “mask” for ${\varvec{A}}$ which can be seen in Fig. 3.

To illustrate Eq. (14), it helps to look at a specific example. E.g., the transition probability from state $${\varvec{\xi}}_{5} = \left( {\begin{array}{*{20}c} 0 & 1 & 0 & 0 \\ \end{array} } \right)$$ to state $${\varvec{\xi}}_{7} = \left( {\begin{array}{*{20}c} 0 & 1 & 1 & 0 \\ \end{array} } \right)$$, which represents starting with involvement only in LNL II and asking for the probability that LNL III becomes involved as well over the next time-step:

16 $$\begin{aligned}
P_{HMM} &\left( \boldsymbol{X}[t + 1] = \boldsymbol{\xi}_7 \mid \boldsymbol{X}[t] = \boldsymbol{\xi}_5 \right) \\
= \, &Q \left( X_1[t+1] = 0; X_1[t] = 0 \right) P_{BN} \left( X_1[t+1] = 0 \mid \tilde{b}_1 \right)^1 \\
\cdot \, &Q \left( X_2[t+1] = 1; X_2[t] = 1 \right) P_{BN} \left( X_2[t+1] = 1 \mid X_1[t] = 0, \tilde{b}_1 \right)^0 \\
\cdot \, &Q \left( X_3[t+1] = 1; X_3[t] = 0 \right) P_{BN} \left( X_3[t+1] = 1 \mid X_2[t] = 1, \tilde{b}_1 \right)^1 \\
\cdot \, &Q \left( X_4[t+1] = 0; X_4[t] = 0 \right) P_{BN} \left( X_4[t+1] = 0 \mid X_3[t] = 0, \tilde{b}_1 \right)^1 \\
= \, &(1 - \tilde{b}_1) \cdot 1 \cdot (\tilde{b}_3 + \tilde{t}_{23} - \tilde{b}_3 \tilde{t}_{23}) \cdot (1 - \tilde{b}_4)
\end{aligned}$$

The interpretation of the last line is that this is the probability that LNL I and IV do not become involved, while LNL III gets infected through lymphatic drainage from either the main tumor or LNL II. The probability of LNL II remaining involved is 1, of course, which is why we take the respective term to the power of 0.

### Marginalization

**Figure 4:**
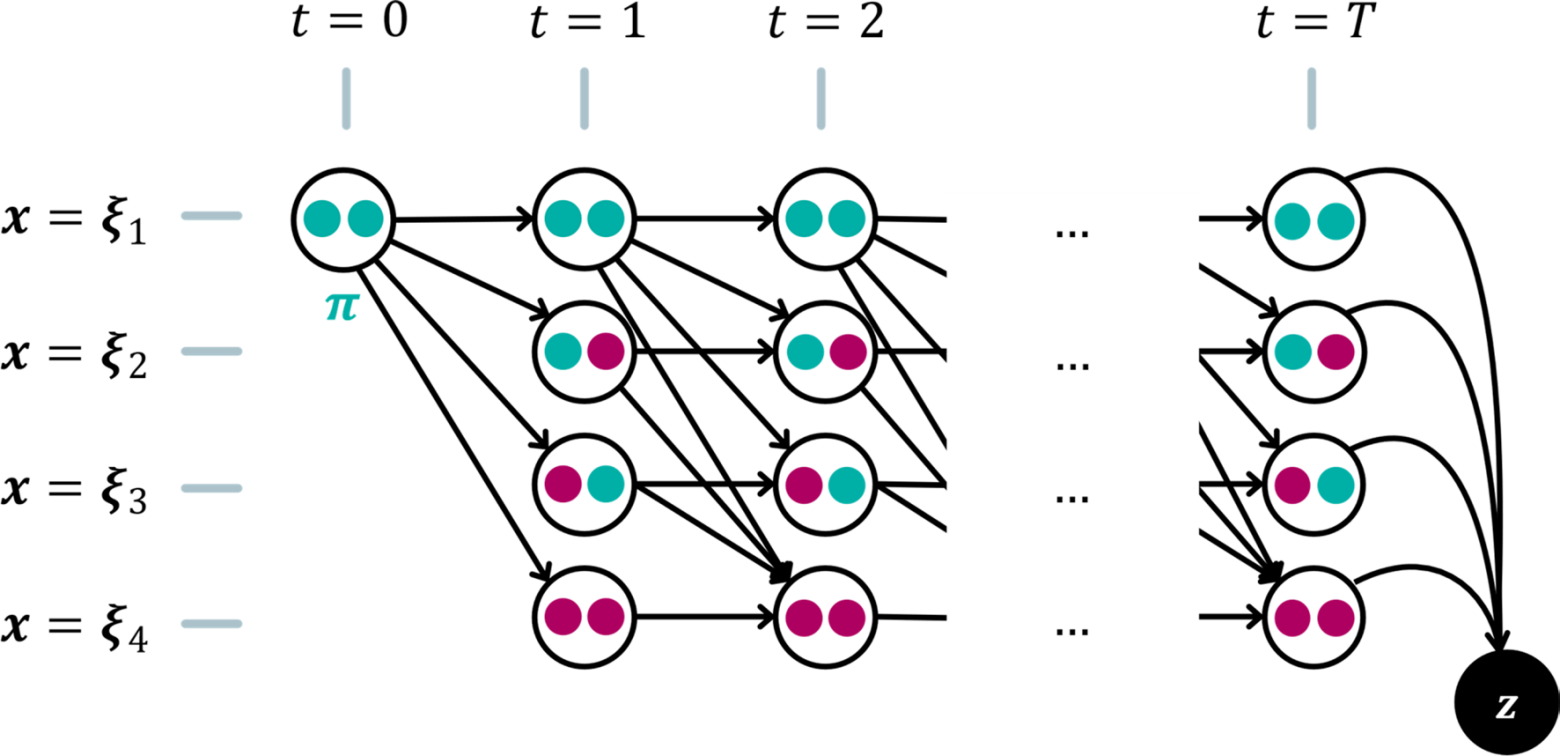
Illustration of possible paths from the starting state ($\pi$) to a diagnosis ($z$) at time T on a discrete grid of time vs state. Only 4 states (corresponding to 2 LNLs) are shown, where green indicates healthy and purple involved. Following the arrows from $\pi$ to $z$ yields a possible path. Some connections between states are forbidden due to $Q$ (no self-healing). To calculate the probability of a diagnosis ($z$), we must marginalize over all paths.

To calculate the likelihood function, we have to calculate the probability of a given diagnostic observation. To that end, we first calculate the probability of observing a given diagnosis ${\varvec{z}} = {\varvec{\zeta}}_{j}$ at a fixed time-step $t$. As depicted in Fig. 4, we must consider every possible evolution of a patient’s disease that leads to the observed diagnosis. Mathematically, this means that we need to marginalize over all such paths. And here is where the HMM-formalism comes in very useful, because this marginalization happens automatically when we multiply the transition matrix with itself:

17 $$\begin{array}{*{20}c} {P\left( {{\varvec{z}} = {\varvec{\zeta}}_{j} ,t} \right) = \left[ {{\varvec{\pi}}^{ \top } \cdot \left( {\varvec{A}} \right)^{t} \cdot {\varvec{B}}} \right]_{j} } \\ \end{array}$$

where the ${\varvec{\pi}}$ is the column vector for the healthy starting state. ${\varvec{A}}$ is multiplied with itself $t$ times and thereby produces a matrix that describes the transition probability from the healthy state to all possible states ${\varvec{x}}\left[ t \right]$ in exactly $t$ time-steps marginalized over the actual pathway of the patient’s disease. The index $\left[ \cdots \right]_{j}$ here means that from the resulting (row-)vector of probabilities we take the component that corresponds to the diagnose ${\varvec{z}} = {\varvec{\zeta}}_{j}$.

The problem that the number of time-steps until diagnosis is unknown cannot be solved in such an elegant fashion. Therefore, we must resort to brute force marginalization and introduce a prior $p\left( t \right)$, which is a discrete distribution over a finite number of time-steps. It describes the prior probability that a patient's cancer is diagnosed at a particular time-step $t$. To get the probability of a diagnosis ${\varvec{z}}$ we must compute

18 $$P \left( \boldsymbol{z} = \boldsymbol{\zeta}_j \right) = \sum_{t \in \mathbb{T}}{ p(t) \cdot P \left( \boldsymbol{z} = \boldsymbol{\zeta}_j , t \right) } = \left[ \sum_{t \in \mathbb{T}}{ p(t) \cdot \boldsymbol{\pi}^{\top} \cdot (\boldsymbol{A})^t \cdot \boldsymbol{B} } \right]_j$$

While the choice of the time-prior may seem unclear at this point, its role for including T-category into this model will be discussed in "Incorporation of T-categoryIncorporation of T-category" section and its choice is discussed in detail in the results section below.

### Inference of model parameters

In the formalism of the last sections, the $P_{HMM}$ depends implicitly through $P_{BN}$ on parameters $\theta = \left\{ {\tilde{t}_{pv} ,\tilde{b}_{v} v \in V,p \in {\text{pa}}\left( v \right)} \right\}$, which—as mentioned—are now probability *rates* and have therefore a slightly different interpretation. Due to the marginalization over time-steps in Eq. (18) the likelihood function additionally depends on the choice and parametrization of the prior $p\left( t \right)$. The parameters are to be inferred from a dataset of lymphatic progression patterns in a cohort of patients. We assume that for each patient we record for every LNL $v$ whether it is involved according to diagnostic modality $k$. In other words, for each patient we observe one of the $V \cdot \left| {\mathcal{O}} \right|$ possible diagnoses. Formally, we can then express the dataset ${\mathbf{\mathcal{Z}}}$ as vector ${\varvec{f}}$ of the number of patients $f_{i}$ for which the diagnosis corresponds to the observational state ${\varvec{\zeta}}_{i}$. The likelihood $P\left( {{\mathbf{\mathcal{Z}}}{|}\theta } \right)$ of observing this dataset, given a particular choice of parameters is then given by

19 $$P \left( \mathcal{Z} \mid \theta \right) = \prod_{i=1}^{V \cdot |\mathcal{O}|}{ P \left( \boldsymbol{\zeta}_i \mid \theta \right)^{f_i} }$$

with the probability $P\left( {{\varvec{\zeta}}_{i} |\theta } \right)$ specified by Eq. (18). The product runs formally over all possible observational states. In reality, $f_{i}$ will be zero for very unlikely configurations of lymph node involvement that are never observed.

By Bayes’ rule, the posterior distribution of those parameters is

20 $$P \left( \theta \mid \mathcal{Z} \right) = \frac{ P \left( \mathcal{Z} \mid \theta \right) P(\theta) }  { \int{ P \left( \mathcal{Z} \mid \theta' \right) P(\theta') d\theta' } }$$

where $P\left( \theta \right)$ is the prior over these parameters. Since they are exclusively probability rates, they must all come from the simplex ${\mathcal{S}} = \left[ {0,1} \right]$. In this work we will choose the most uninformative prior

21 $$P(\theta) = 
\begin{cases}
    1 & \text{if} \quad \theta \in \mathcal{S}^{V(V-1)} \\
    0 & \text{otherwise}
\end{cases}$$

While it is easy to compute the likelihood, it is not feasible to efficiently calculate the normalization constant in the denominator of Eq. (20). Hence, we will use Markov-chain Monte Carlo sampling methods to estimate the parameters $\theta$ and their uncertainty.

### Incorporation of T-category

We have introduced the hidden Markov model with the promise that it could handle the concept of T-categories through its explicit modelling of dynamic processes. To keep up with that, we will now explain how this is achieved using the time-prior $p\left( t \right)$.

The core idea is to assume that early T-category and late T-category tumors share the same patterns of metastatic progression, except that late T-category tumors are on average diagnosed at a later point in time, and thereby also show, on average, higher LNL involvement. Formally, this can be described by assuming a different time-prior $p_{T} \left( t \right)$ for every T-category. On the other hand, the transition matrix ${\varvec{A}}$ is assumed to be the same for all T-categories.

For the inference of model parameters, the training data is split into subgroups according to T-category. We now define a column-vector ${\varvec{f}}_{T}$ separately for each T-category, which counts the number of patients in the dataset that were diagnosed with one of the possible observational states and a given T-category. The log-likelihood from which we want to sample is then simply a sum of the likelihoods as above, where the essential difference is that we equip each marginalization over time with a different time-prior $p_{T} \left( t \right)$, according to its T-category:

22 $$\log{P \left( \mathcal{Z} \mid \theta \right)} = \sum_{T=1}^4{ \log{ \left[ \sum_{t \in \mathbb{T}}{ p_T(t) \cdot \boldsymbol{\pi}^{\top} \cdot (\boldsymbol{A})^t \cdot \boldsymbol{B} } \right] } \cdot \boldsymbol{f}_T }$$

The logarithm must be taken elementwise for the resulting row-vector inside the square brackets. The only data-dependent term here is the vector ${\varvec{f}}_{T}$ counting the occurrences of all possible observations. It is again important to note that the only difference between the part of the log-likelihood for the different T-categories is the exact shape or parametrization of the time-prior. The transition probabilities, and hence also the transition matrix $A$, are the same for all T-categories. For this to work, we rely on the assumption that different typical patterns of nodal involvement for the same primary tumor location are caused mainly by different progression times.

### Sampling

For learning we employed the python implementation of an advanced ensemble sampler called emcee^34^ based on an affine invariant ensemble sampler^36^ to draw parameter samples from the likelihood in Eq. (22). Although sampling is the slowest and least preferable option of inference it is also without doubt in a large number of cases the only available option and in our case even feasible; we get relatively short autocorrelation times (around a couple of hundred steps) and an average modern multicore CPU can easily draw hundreds of thousands of samples within minutes.

**Figure 5:**
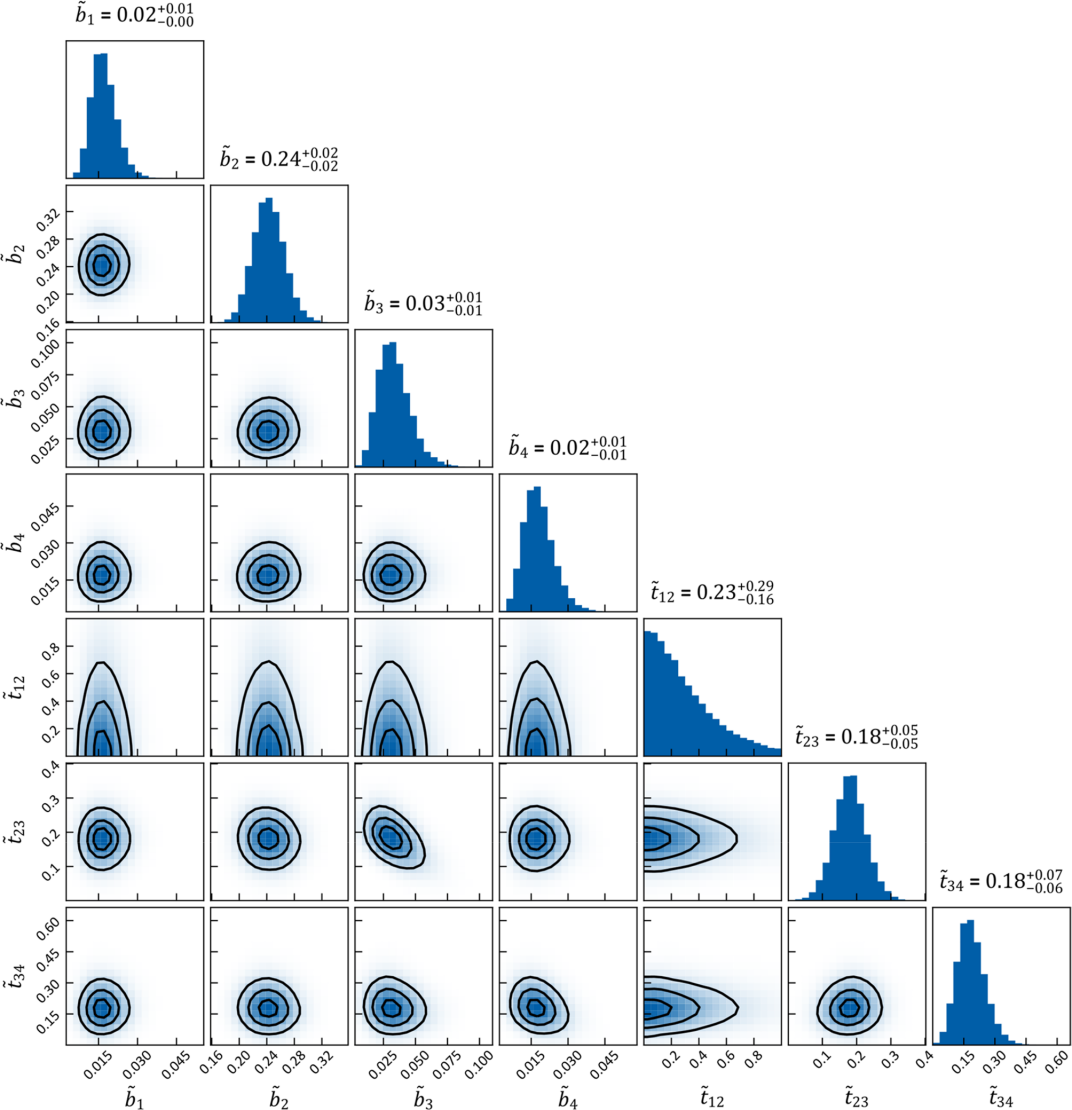
Corner plot of the sampled parameters for the HMM model parameters. The histograms on the diagonal show the 1D marginals, while the lower triangle shows all possible combinations of 2D marginals. The black lines are the isolines enclosing 20%, 50% and 80% of the sampled points respectively. Correlations between the parameters can at most be seen between $\tilde{t}_{23}$ and $\tilde{b}_{3}$.

Many distributions in the form of histograms we show in this work are made by computing the respective quantity—e.g., the risk (see below)—for a subset of the sampled parameters. We typically randomly select between 1 and 2% of the 200,000 samples drawn after the so-called *burn-in* phase, when the sampling has already converged to the target distribution, as a subset. The learned parameter densities are depicted as a corner^37^ plot (e.g. in Fig. 5), which is further discussed in "Application to oropharyngeal HNSCC" section.

### Risk assessment of microscopic involvement

With the a parameter set $\theta = \left( {\left\{ {\tilde{b}_{v} } \right\},\left\{ {\tilde{t}_{{{\text{pa}}\left( v \right)v}} } \right\}} \right)$, we can assess the risk of nodal involvement, given a diagnosis ${\varvec{z}}$, of a new patient. Using Bayes’ law, the risk for a certain LNL $v$ being involved is given by the conditional probability

23 $$R \left( X_v = 1 \mid \boldsymbol{z}, \theta \right) = \frac{ P \left( \boldsymbol{Z} = \boldsymbol{z} \mid X_v = 1, \theta \right) P \left( X_v = 1 \mid \theta \right) }{ P \left( \boldsymbol{Z} = \boldsymbol{z} \mid \theta \right) }
= \frac{ \sum_{\left\{ i \colon \xi_{iv} = 1 \right\}}{ P \left( \boldsymbol{Z} = \boldsymbol{z} \mid \boldsymbol{\xi}_i, \theta \right) P \left( \boldsymbol{\xi}_i \mid \theta \right) } }{ P \left( \boldsymbol{Z} = \boldsymbol{z} \mid \theta \right) }$$

Note that on the right, we have explicitly written out the marginalization over all hidden states ${\varvec{\xi}}_{i}$ that have LNL $v$ involved. We have written the state of LNL $v$ in the state ${\varvec{\xi}}_{i}$ as $\xi_{iv}$. The denominator can be computed using Eq. (18), which already includes the marginalization over all hidden states ${\varvec{\xi}}_{i} .$

The process of sampling randomly generates $L$ sets of parameters $${\varvec{\theta}} = \left( {\begin{array}{*{20}c} {\theta_{1} } & {\theta_{2} } & \ldots & {\theta_{L} } \\ \end{array} } \right)$$. They are therefore random variables and so is the risk $R\left( {X_{v} |{\varvec{z}},\theta } \right)$ since it is a function of $\theta$. Using the Monte Carlo estimator, we can therefore compute the moments of the distribution over the risk, including e.g. the expectation value

24 $$\mathbb{E}_{\boldsymbol{\theta}} \left[ R \left( X_v = 1 \mid \boldsymbol{z} \right) \right] = \frac{1}{L} \sum_{k = 1}^L{ R \left( X_v = 1 \mid \boldsymbol{z}, \theta_k \right) }$$

In the result sections below, we compute the individual risks for a large enough number $L$ of sampled parameters. Thereby, we can compute histograms for the risk that will approach the real probability density of the respective risk for $L \to \infty$. This provides additional information on the uncertainty in the predicted risk resulting from uncertainty in the model parameters.

### Learning and risk assessment for incomplete diagnoses

A diagnosis is often not complete, meaning that not all LNLs might have been observed with all available diagnostic modalities. E.g., while a patient may have undergone a PET-CT scan to identify suspicious lymph nodes in the whole neck, FNA is only performed in a subset of LNLs. Hence, we must be able to deal with “incomplete” observations for some LNLs. To do so, we first introduce a new observation variable

25 $$\begin{array}{*{20}c} {d_{v}^{{\mathcal{O}}} \in \left\{ {0,1,\emptyset } \right\}} \\ \end{array}$$

where $\emptyset$ indicates *unobserved*. One way to do this is to introduce a *match function*

26 $$\operatorname{match}(\boldsymbol{d},\boldsymbol{z}) :=
\begin{cases}
    \text{true} & \text{if} \quad d_v^{\mathcal{O}} = z_v^{\mathcal{O}} \,\lor\, d_v^{\mathcal{O}} = \emptyset; \quad \forall v, \mathcal{O} \\
    \text{false} & \text{otherwise}
\end{cases}$$

which returns *true* if a—potentially incomplete—diagnosis ${\varvec{d}}$ is consistent with a complete observation ${\varvec{z}}$. We will use this function for conveniently marginalizing over the missing observations. In analogy to Eq. (23), we can compute the risk for an incomplete observation as

27 $$R \left( X_v = 1 \mid \boldsymbol{z}, \theta \right) = \frac{ P \left( \boldsymbol{d} \mid X_v = 1, \theta \right) P \left( X_v = 1 \mid \theta \right) }{ P \left( \boldsymbol{d} \mid \theta \right) }
= \frac{ \sum_{\left\{ i \colon \xi_{iv} = 1 \right\}}{ P \left( \boldsymbol{d} \mid \boldsymbol{\xi}_i, \theta \right) P \left( \boldsymbol{\xi}_i \mid \theta \right) } }{ P \left( \boldsymbol{d} \mid \theta \right) }$$

The terms in the enumerator on the right-hand side are given by:

28 $$\begin{aligned} P\left( {{\varvec{d}}\,|\,{\varvec{\xi}}_{i} ,\theta } \right)P\left( {{\varvec{\xi}}_{i} \,|\,\theta } \right) & = \mathop \sum \limits_{{\left\{ {{\varvec{\zeta}}_{j} :{\text{match}}\left( {{\varvec{d}},{\varvec{\zeta}}_{j} } \right)} \right\}}} P\left( {{\varvec{\zeta}}_{j} \,|\,{\varvec{\xi}}_{i} ,\theta } \right)P\left( {{\varvec{\xi}}_{i} \,|\,\theta } \right) \\ & = \mathop \sum \limits_{{\left\{ {j:{\text{match}}\left( {{\varvec{d}},{\varvec{\zeta}}_{j} } \right)} \right\}}} b_{ij} \left[ {\mathop \sum \limits_{{t \in {\mathbb{T}}}} p_{T} \left( t \right) \cdot {\varvec{\pi}}^{ \top } \cdot \left( {\varvec{A}} \right)^{t} } \right]_{i} \\ \end{aligned}$$

In this case, $b_{ij}$ denotes the element of the observation matrix that corresponds to state ${\varvec{\xi}}_{i}$ and observation ${\varvec{\zeta}}_{j}$. Again, the indices $\left\{ {i \, | \, \xi_{iv} = 1} \right\}$ correspond to all possible states with a positive involvement in lymph node level $X_{v}$. Essentially, the whole term is the likelihood of an observation ${\varvec{d}}$ where we have just removed all entries that correspond to states with $X_{v} \ne 1$ both from the observation matrix and the resulting probability vector of the evolution. It can therefore be easily computed algebraically again.

The evidence in the denominator becomes essentially a marginalization over all possible diagnoses that are not available to us or that we deem unimportant

29 $$P \left( \boldsymbol{d} \mid \theta \right) = \sum_{\left\{ j \colon \operatorname{match}(\boldsymbol{d}, \boldsymbol{\zeta}_j) \right\}}{ \left[ \sum_{t \in \mathbb{T}}{ p_T(t) \cdot \boldsymbol{\pi} \cdot (\boldsymbol{A})^t \cdot \boldsymbol{B} } \right]_j }$$

We can make this summation a bit more elegant using a column-vector ${\varvec{c}}^{{\varvec{d}}}$ that has entries corresponding to the match-function

30 $$\begin{array}{*{20}c} {c_{i}^{{\varvec{d}}} = {\text{match}}\left( {{\varvec{d}},{\varvec{\zeta}}_{i} } \right)} \\ \end{array}$$

where every *true* corresponds to a 1 and every *false* to a 0. This way we can rewrite Eq. (29) in the following way:

31 $$P \left( \boldsymbol{d} \mid \theta \right) = \sum_{t \in \mathbb{T}}{ p_T(t) \cdot \boldsymbol{\pi} \cdot (\boldsymbol{A})^t \cdot \boldsymbol{B} \cdot \boldsymbol{c}^{\boldsymbol{d}}}$$

Using this algebraic notation for marginalizing over unknown or incomplete observations also allows us to encode whole datasets $${\mathbf{\mathcal{D}}} = \left( {\begin{array}{*{20}c} {{\varvec{d}}_{1} } & {{\varvec{d}}_{2} } & \cdots & {{\varvec{d}}_{N} } \\ \end{array} } \right)$$ of (potentially incomplete) observations in the form of matrices

32 $$\begin{array}{*{20}c} {C = \left( {\begin{array}{*{20}c} {{\varvec{c}}^{{{\varvec{d}}_{1} }} } & {{\varvec{c}}^{{{\varvec{d}}_{2} }} } & \cdots & {{\varvec{c}}^{{{\varvec{d}}_{N} }} } \\ \end{array} } \right)} \\ \end{array}$$

## Application to oropharyngeal HNSCC

We considered the graph in Fig. 1 as the underlying abstract representation of the lymphatic flow with only one diagnostic modality per LNL. Just as (Pouymayou et al.)^31^, we used the reconstructed dataset of early T-category patients with oropharyngeal carcinomas detailing ipsilateral nodal involvement of the LNLs I to IV from (Sanguineti et al.)^8^ for inference. Because this publication only reported on 103 $N_{ + }$ patients, we added 44 $N_{0}$ entries to reflect that around 30% of early T-category patients with pharyngeal HNSCC are observed to be node negative^15^. To make this paper self-contained, the dataset is provided in the supplementary information. During training of the HMM we fixed both sensitivity and specificity to 1, since we assumed the pathological report to be the ground truth. For the subsequent risk assessment, we set the sensitivity to $s_{N} = 81\%$ and the specificity to $s_{P} = 76\%$, which represent values for CT imaging^35^ analogous to the work on BN^31^.

**Figure 6:**
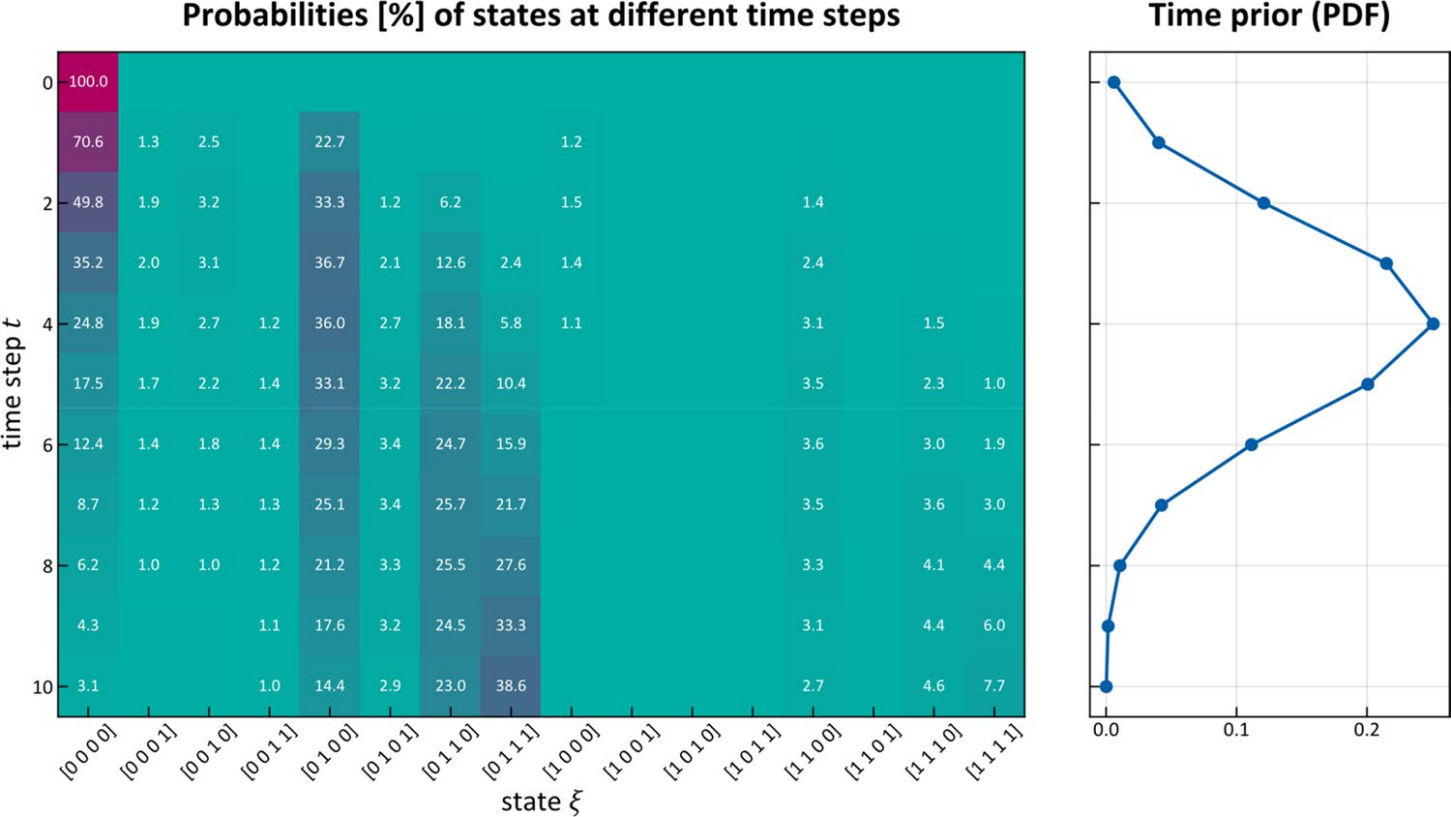
Probability of being in each hidden state as a function of time (left). The color indicates low (green) and high (red) probabilities, which are also written on the respective pixel in percent if larger than 1%. We used the mean of the inferred parameter samples to compute the probabilities. On the right, the used time-prior is plotted with which each column on the left will be weighted.

For the time-prior $p\left( t \right)$ we chose a Binomial distribution illustrated in Fig. 6 (right) because it has finite support, its mean can be controlled by one parameter $p$, and its shape reflects the intuitive assumption that the probability of diagnosing a patient with cancer is small for very early time-steps (when the tumor is small) and very late time-steps (when a patient’s symptoms are so severe that it is unlikely that they did not notice their disease earlier). The number of time-steps was fixed to 10 and the parameter $p$ was set to $p = 0.4$ for early T-category patients, meaning that the probability of diagnosis peaks around $t = 4$, but is non-zero for earlier or later times. While it is important to have enough time-steps so the system can evolve, it can be shown that the results presented below are mostly independent of the exact choice of the time-prior shape and the number of time-steps. This is further detailed in Appendix A.2.

### A patient’s evolution

**Figure 7:**
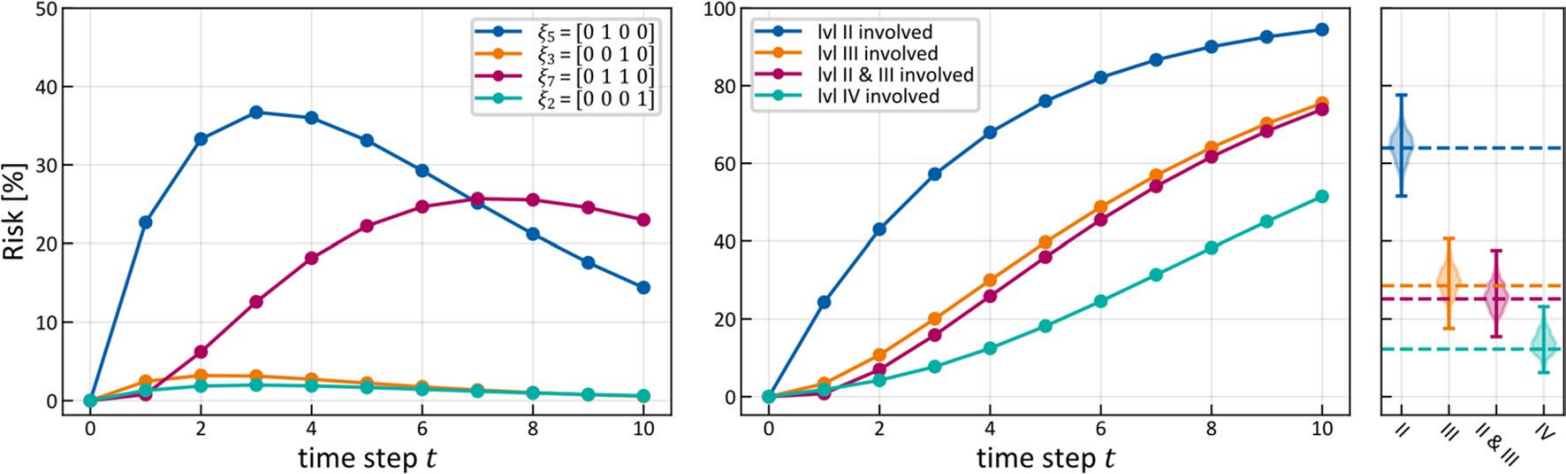
(left) Probability of certain hidden state vs time; (middle) Probability of LNL’s involvement marginalized over the other LNL’s involvement vs time; (right) The same probabilities as in the middle, but also marginalized over the time-prior and depicted as violin plots. The dashed lines represent the prevalence in the dataset^8^ that was used for training.

Having inferred the parameters $\tilde{t}_{{{\text{pa}}\left( v \right)v}}$ and $\tilde{b}_{v}$, we can model how the state of LNL involvement evolves over the time-steps that support the chosen prior. In Figs. 6 and 7 (left), we have plotted the probability of each hidden state ${\varvec{\xi}}_{i}$ for each time-step (calculated for the mean over all parameter samples). At time-step zero the patient is healthy, and the system is by definition in the initial state with probability 1. One time-step later the individual lymph nodes are involved with the base probability rates $\tilde{b}_{v}$ (Fig. 5).

For example, after one time-step the state $${\varvec{\xi}}_{5} = \left[ {\begin{array}{*{20}c} 0 & 1 & 0 & 0 \\ \end{array} } \right]$$ has a probability of involvement of $P\left( {{\varvec{X}}\left[ 1 \right] = {\varvec{\xi}}_{5} \, | \, {\varvec{X}}\left[ 0 \right] = {\varvec{\pi}}} \right) \approx 22.7\%$ while the respective base probability rate is $\tilde{b}_{2} \approx 24\%$. They are not quite the same, since state ${\varvec{\xi}}_{5}$ is only one of the eight states that include involvement of LNL II. After the first time-step, the transmission between the LNLs starts to play a role. From $t = 2$ onwards, we can e.g. see an increase in the joint involvement of LNL II and III $${\varvec{\xi}}_{7} = \left[ {\begin{array}{*{20}c} 0 & 1 & 1 & 0 \\ \end{array} } \right]$$ whereas the probability of involvement in LNL III only ($${\varvec{\xi}}_{3} = \left[ {\begin{array}{*{20}c} 0 & 0 & 1 & 0 \\ \end{array} } \right]$$) is low. In Fig. 5, this corresponds to a high rate of spread from level II to III ($\tilde{t}_{23} \approx 18\%$), since the base probability rate for level III is rather low ($\tilde{b}_{3} \approx 3\%$). After the tenth time-step, we find state $${\varvec{\xi}}_{8} = \left[ {\begin{array}{*{20}c} 0 & 1 & 1 & 1 \\ \end{array} } \right]$$, representing the involvement of the whole lymphatic chain from LNL II down to LNL IV, to be the most likely state. If we would continue to evolve the system beyond this time-step, we would find that the probability of the final and worst state $${\varvec{\xi}}_{16} = \left[ {\begin{array}{*{20}c} 1 & 1 & 1 & 1 \\ \end{array} } \right]$$ grows to 1 for $t \to \infty$. However, this occurs at a time much later than the typical time of diagnosis.

In contrast to the probability of hidden states, the probability of a single LNL’s involvement can only increase over time, as depicted in Fig. 7 (middle), since it is a marginalization of all the eight states that contain the respective LNL’s involvement. One of these eight states is always the final state ${\varvec{\xi}}_{16}$ and hence the probability for involvement in any LNL must approach 1 for increasing $t$. Intuitively, this naturally arises as every time-step harbors the risk of a level becoming involved, while self-healing is forbidden.

Finally, in the right panel of Fig. 7 shows the probability of a LNL’s involvement marginalized over all time-steps using the time-prior. The probabilities plotted in this window are the result of marginalizations of the matrix plotted in Fig. 6: first, selectively along the x-axis; and secondly, weighted along the y-axis. These marginalized probabilities are compared to the prevalence of LNL involvement in the dataset used during learning. The agreement between our model and the data observed in Fig. 7 (right) verifies that the model can accurately describe the data.

### Risk prediction and comparison to BN model

**Figure 8:**
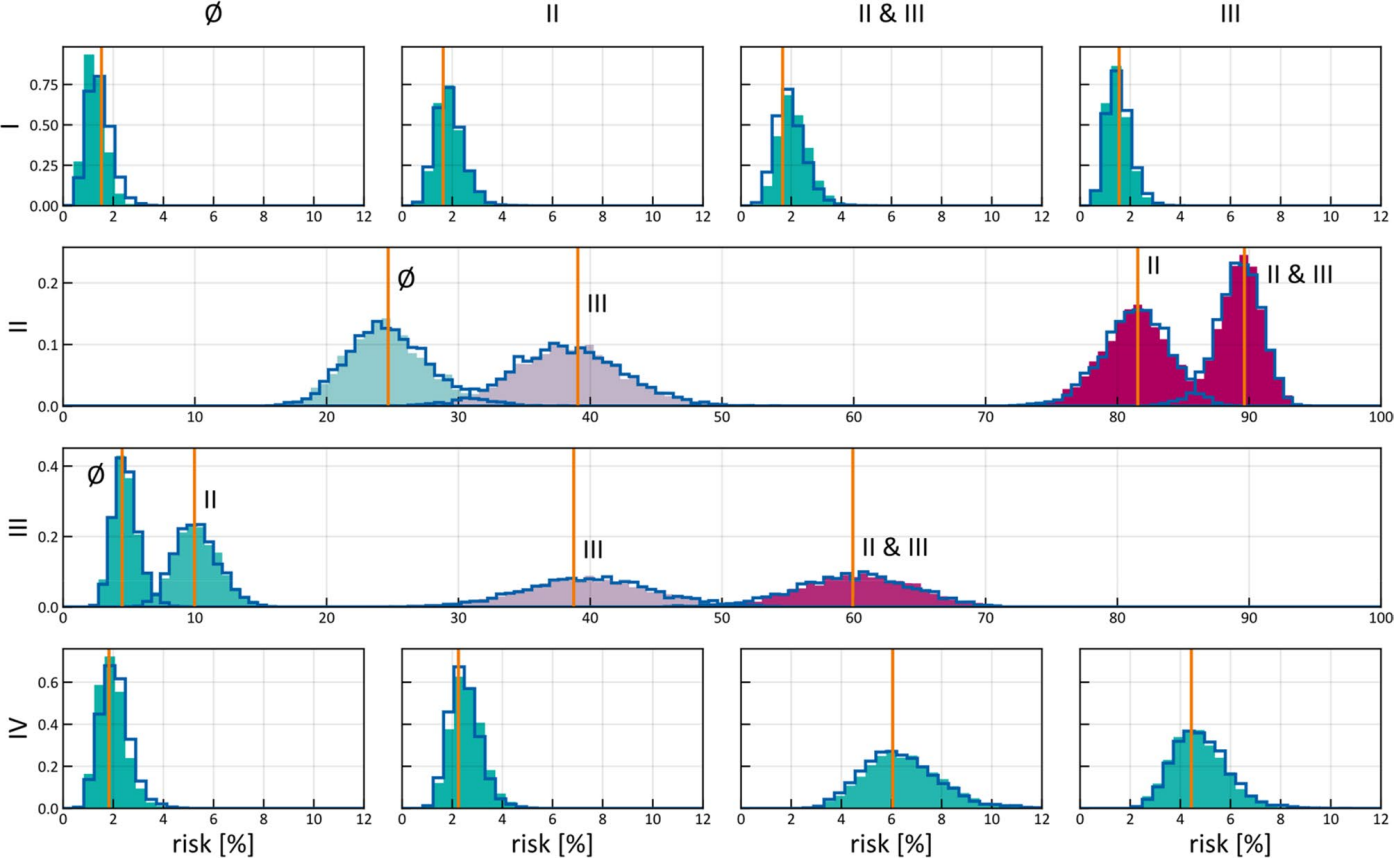
Risk assessment for the involvement of different LNLs (rows), given positive observational findings in specified LNLs (columns or labels next to histograms). E.g. row 3 depicts the risk of involvement in LNL III, given different observed involvements (from left to right: no involvement, LNL II only, LNL III only, and LNL II and III but no others). The orange line depicts the maximum likelihood result from (Pouymayou et al.)^31^, the blue outline histogram represents the BN sampling solutions and the solid coloured histograms are the results from the HMM. The colour goes from green (low risk) to red (high risk). Of 200,000 parameter samples, 2% were used to create this plot.

In Figs. 6 and 7, we have considered the intrinsic time evolution of the hidden state describing lymphatic progression in the patient population. Now, we calculate the risk of LNL involvement conditioned on a given diagnostic observation as described in "Risk assessment of microscopic involvement" section. Figure 8 shows the estimated risk of involvement for four possible observational states. The risk is shown in the form of a histogram, which is obtained by taking a random subset of the sampled parameters (we took 2% of 200,000 samples) and computing the risk for each sample as explained in "Risk assessment of microscopic involvement" section.

As LNL II is involved in the majority of patients, the probability of involvement is high even for negative imaging findings ($\approx 25\%$ for $N_{0}$ patients). Positive imaging findings of involvement in level III further increases the risk for metastases in level II to almost 40% since the main cause of LNL III’s involvement is the spread from an already involved level II. Vice versa, the risk in level III doubles from around 5% for $N_{0}$ patients to approximately 10% when II is diagnosed with metastases. But we can also observe this correlation the other way around: If the CT image indicates involvement in LNL III, but not in II, then there is actually a 60% chance that this has been a false positive finding, considering how rarely level III alone is involved. Finally, also the risk of involvement in level IV is increased from 2 to 4% and 6% when observing metastases in level III or in both level II and III, respectively. It is important to note that these predictions do not only depend on the dataset that was used to train the model, but also on the sensitivity and specificity used to produce a new patient’s diagnosis.

It can be seen that the risk for involvement in level I is low, regardless of diagnostic findings in the levels II and/or III. This is because the base probability rate $\tilde{b}_{1} \approx 2\%$ is very small and there is no other LNL that drains into this one. Because level I is metastatic so rarely, involvement of level II is dominated by the base probability rate $\tilde{b}_{2}$ while the probability of spread from level I to II is almost inconsequential. This leads to the very broad distribution over the transmission probability $\tilde{t}_{12}$ seen in Fig. 5 as almost any value of $\tilde{t}_{12}$ is consistent with the data.

Figure 8 also compares risk estimation for HMM-based model to the previously published BN model^31^ described in "Bayesian network of lymph node level involvement" section. To that end, parameters of the BN model have been sampled from the likelihood function (6). The histograms of estimated risk are nearly identical, which verifies that the HMM-based model and the BN-based model yield the same risk predictions—a feature which is expected from the HMM when only considering a single T-category and thus no time information is present. Figure 8 further shows that risk predictions of the BN model using the maximum likelihood estimators of its parameters^31^, agree with the mean of the histogram. However, the sampling method presented here has the additional advantage over previously published model that if provides confidence intervals for the predicted risk.

### Risk prediction for later T-category

To illustrate the capability of the model to incorporate T-category into the risk prediction via the time-prior, we increased the parameter $p$ in the Binomial distribution while keeping the learned parameters $\tilde{b},\tilde{t}$ from the previous section, which were inferred from a dataset of early T-category patients.

**Figure 9:**
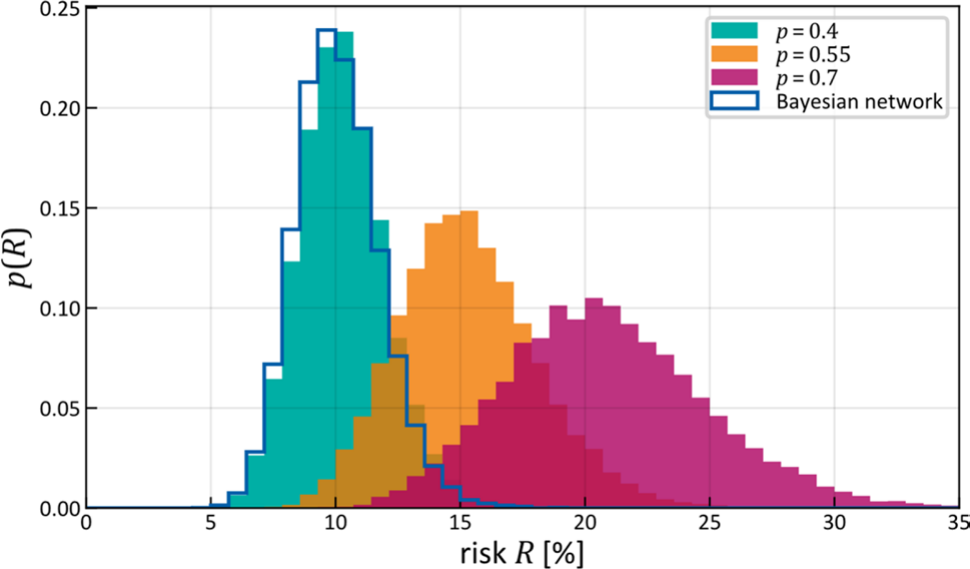
Risk prediction for LNL III, given observed positive involvement in LNL II and negative observations in all other LNLs (assuming $s_{N} = 81\%$ and $s_{P} = 76\%$)^35^. The Binomial parameter $p$ was fixed to $0.4$ for parameter learning (green), representing early T-category patients. Increasing this parameter results in higher risk. The blue outline shows the risk in level III obtained for the Bayesian network model. The histograms correspond to 1% of the 200,000 samples.

In Fig. 9 we consider the risk of microscopic involvement in LNL III, given observed positive involvement only in LNL II and negative observations in all other LNLs. Increasing the mean of the time-prior yields higher risk of microscopic involvement. This makes intuitive sense since the expected number of time-steps between healthy state and diagnose increases, and therefore the probability of being in a more involved hidden state. Consequently, also the predicted risk of microscopic involvement despite negative diagnostic observation increases (orange and red histograms in Fig. 9). The variance of that risk increases as well, since predictions typically become more and more uncertain the further one extrapolates into the future. This shows that the principal idea behind the choice of an HMM works as intended.

### Time-prior learning

Although we have now shown how shifting the mass of the time prior towards later time steps generally increases the risk of involvement, we are not yet able to identify the different T-categories with certain time prior distributions. Throughout this work we will continue to use the Binomial distribution as time prior. But even with this simplifying choice, the question remains: Which Binomial parameter should one choose for the different T-categories? Our approach to this issue is to fix the Binomial time prior’s $p$ parameter for one T-category and simultaneously learn the transition matrix parameters $\tilde{b}_{v}$ and $\tilde{t}_{{{\text{pa}}\left( v \right)v}}$ together with the Binomial time prior’s $p$ parameters for all other T-categories based on the likelihood function (22) described in "Incorporation of T-category" section. If we do not fix the time prior parameter for any T-category, the system becomes overdetermined and very strong correlations between the spread parameters and the Binomial parameter appear (see also Appendix A.2). Then, if the model is presented with different degrees of involvement at different T-categories it can separate them by shifting the mass of the respective time priors apart, but it will learn the common spread parameters. This approach requires a dataset containing nodal involvement reports for patients with different T-categories.

To the best of our knowledge, the dataset of surgically treated early T-category (T1 and T2) patients in^8^ is the only dataset containing detailed information on LNL involvement, and no corresponding dataset exists for late T-category patients (T3 and T4). However, the literature provides estimates on the ratio of $N_{0}$ (no nodal involvement) and $N_{ + }$ (at least one involved LNL) patients for advanced T-categories. Here, we show that this information is sufficient to estimate the Binomial time prior’s $p$ for late T-category patients. This situation can be considered as a special case of learning from incomplete observations as described in "Learning and risk assessment for incomplete diagnoses" section.

As an example for the simultaneous learning, let us consider a patient database ${\mathbf{\mathcal{Z}}}_{{{\text{early}}}}$ for early T-category patterns of involvement and one for late T-category ${\mathbf{\mathcal{Z}}}_{{{\text{late}}}}$ together with the respective frequency vectors ${\varvec{f}}_{{{\text{early}}}}$ and ${\varvec{f}}_{{{\text{late}}}}$. Then the log-likelihood for combined learning is given by

33 $$\begin{aligned} & {\text{log}}\:P\left( {{\mathbf{\mathcal{Z}}}_{{{\text{early}}}} ,{\mathbf{\mathcal{Z}}}_{{{\text{late}}}} {\, | \,}\theta ,p_{{{\text{late}}}} } \right) \\ & \quad = \log \left[ {\mathop \sum \limits_{t \in 0}^{n} {\mathfrak{B}}\left( {p_{{{\text{early}}}} ,n} \right) \cdot {\varvec{\pi}}^{ \top } \cdot \left( {\varvec{A}} \right)^{t} \cdot {\varvec{B}}} \right] \cdot {\varvec{f}}_{{{\text{early}}}} \\ & \quad \quad + \log \left[ {\mathop \sum \limits_{t \in 0}^{n} {\mathfrak{B}}\left( {p_{{{\text{late}}}} ,n} \right) \cdot {\varvec{\pi}}^{ \top } \cdot \left( {\varvec{A}} \right)^{t} \cdot {\varvec{B}} \cdot {\varvec{C}}} \right] \cdot {\varvec{f}}_{{{\text{late}}}} \\ \end{aligned}$$

where ${\mathfrak{B}}\left( {p,n} \right)$ is the Binomial distribution with parameters $p \in \left[ {0,1} \right]$ and $n \in {\mathbb{N}}$, where the early T-category parameter $p_{{{\text{early}}}}$ (along with the number of time steps $n$) must be fixed beforehand. ${\varvec{C}}$ is a matrix for handling incomplete observations as introduced in "Learning and risk assessment for incomplete diagnoses" section, which in this case is a $\left\{ {0,1} \right\}^{2 \times N}$ matrix

34 $$\begin{array}{*{20}c} {C = \left( {\begin{array}{*{20}c} 1 & 0 \\ 0 & 1 \\ \vdots & \vdots \\ 0 & 1 \\ \end{array} } \right)} \\ \end{array}$$

that marginalizes over all diagnoses that indicate some nodal involvement ($N_{ + }$). The resulting vector after the matrix multiplication with ${\varvec{C}}$ has only two components which correspond to the probability of observing the $N_{0}$ diagnosis and any other diagnosis ($N_{ + }$) respectively.

**Figure 10:**
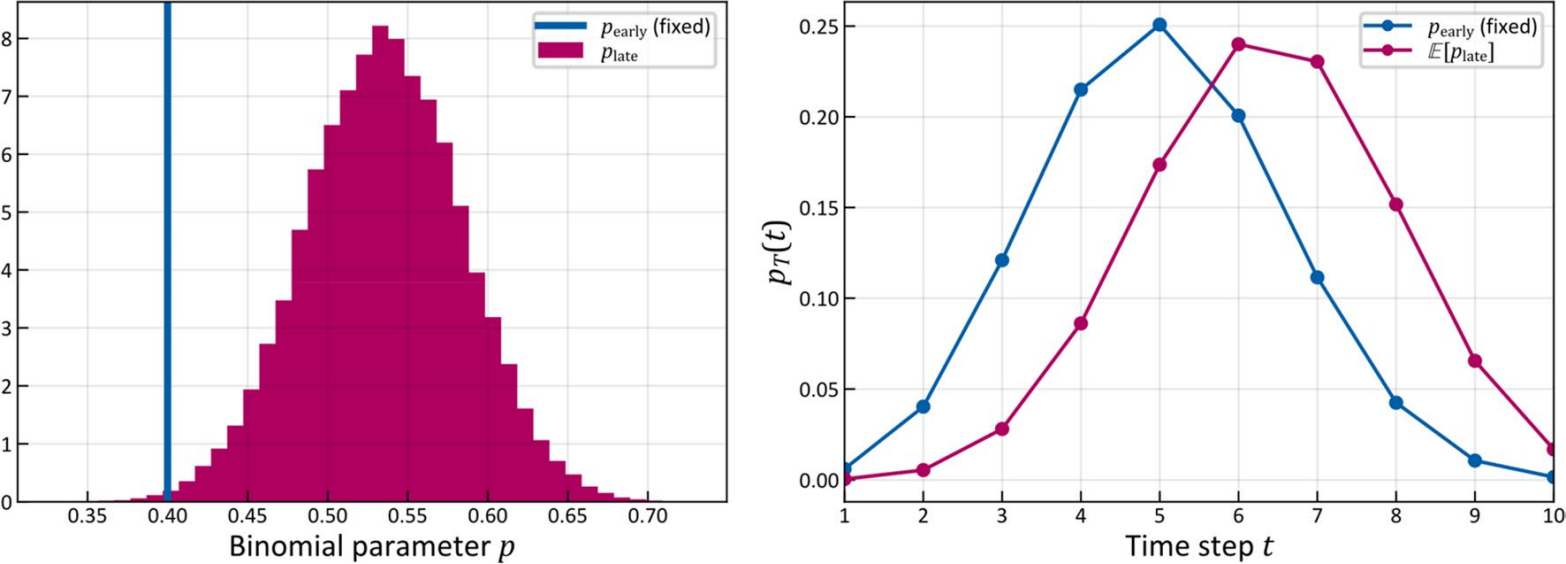
Sampled late T-category p parameter given an early T-category cohort and a fixed fraction of ${\text{N}}_{0}$ patients (20%) for late T-category (left). Plots of the PMFs of the fixed early T-category Binomial distribution and the distribution for the expected value of the late T-category parameter (right).

This approach allows us to infer the spread parameters and the late T-category’s Binomial parameter $p_{{{\text{late}}}}$ if we do not have a database of late T-category patients. Simply the percentage of patients without nodal involvement in addition to an early T-category cohort is enough. We show this in Fig. 10, where we used the same dataset of early T-category patients as in the sections before, but we added the information that for late T-category the $N_{0}$ portion would reduce from 30 to 20%. More specifically, this amounts to creating a second “database” of another 147 patients, but instead of detailed patterns of involvement, each patient has either no nodal involvement (healthy state w.r.t. LNLs) or have some ($${\varvec{f}}_{{{\text{late}}}} = \left( {\begin{array}{*{20}c} {29} & {118} \\ \end{array} } \right)$$). In the latter case, the system marginalizes over all possible observations except the healthy diagnosis. Sensitivity and specificity were kept the same as before. The learned spread parameters $\tilde{b}_{v}$ and $\tilde{t}_{{{\text{pa}}\left( v \right)v}}$ are the same as before, since the sampler is not presented with different patterns of progression, but we additionally infer the parameter $p_{\text{late}}$ of a Binomial distribution representing the late T-category’s time-prior just based on a reduction of the $N_{0}$ portion.

**Figure 11:**
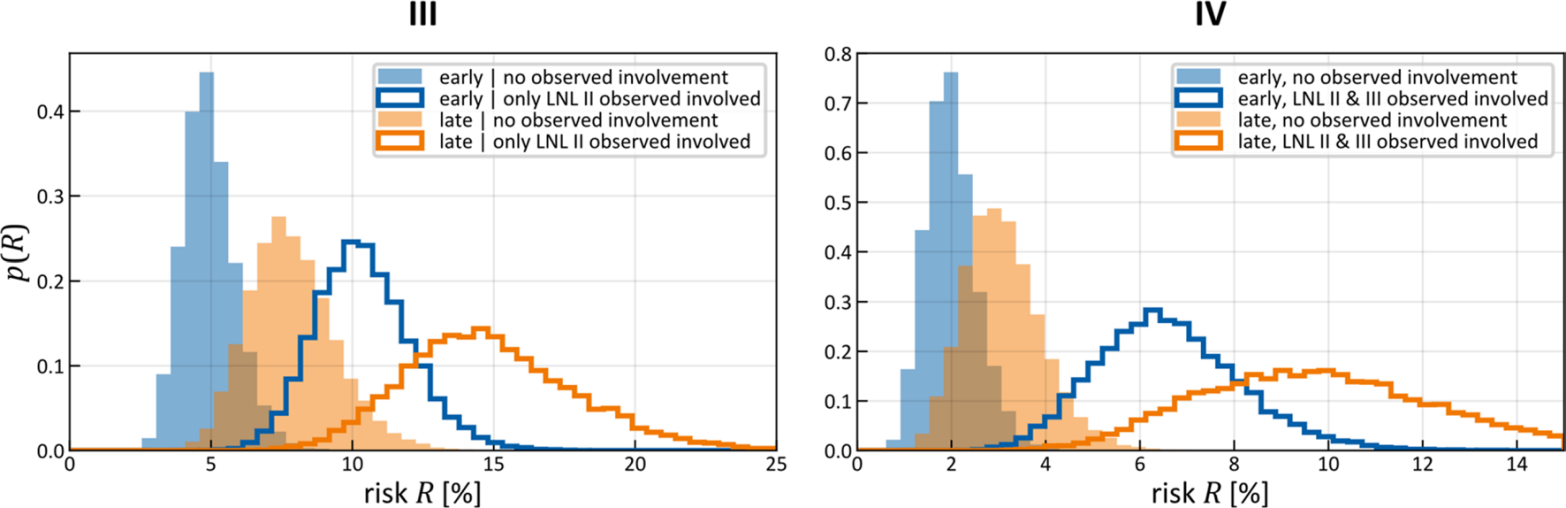
Distributions over risk of involvement for LNL III (left) and LNL IV (right), each for early and late T-category as well as depending on the given observed involvement. The sampled parameters displayed here are a randomly selected subset (1% of 200,000) from simultaneous learning. Comparison with Fig. 8 shows that these predictions still agree with the results from the early stage only learning.

A comparison of the involvement risk for LNLs III and IV for different combinations of early and late T-category given different observed diagnoses is shown in Fig. 11. The risk of microscopic involvement in level III is around 5% for early T-category patients which are observed $N_{0}$. When only level II is observed to harbor metastases, the risk increases to approximately 10%. If, in addition, the patient has late T-category tumor, the risk increases further to 15%. Similarly, the risk of microscopic involvement in level IV is low (~ 2%) for early T-category patients without diagnosed metastases but increases to substantially higher values (~ 10%) for late T-category patients with observed metastases in LNL II and III.

The absolute numbers for risk of involvement depend on the dataset of progression patterns, the fraction of $N_{0}$ patients, and the assumed values for sensitivity and specificity of imaging. Larger datasets are warranted before clinical decisions can be based on the model. Nevertheless, Fig. 11 illustrates the potential of the HMM-based model to personalize microscopic involvement risk based on the individual patient's state of disease progression. For example, one could consider excluding level IV from the elective CTV for early T-category patients without visible metastatic disease in level III.

## Discussion and outlook

In this work, we presented a probabilistic model based on HMM for predicting the lymphatic progression of HNSCC through a patient’s LNLs. The model allows for estimating risk of microscopic LNL involvement, given two patient-specific diagnostic observations: (1) imaging information on the location of macroscopic metastases, and (2) T-category. The first aspect has been addressed in a previous publication, and we showed that the predictions of the new HMM-based model agree with the previously published BN-based model when given the same training data. The HMM-based model adds the capability to include T-category into the assessment of LNL involvement risk by modeling the transitions between different states of nodal progression over discrete time-steps. This assumes that for a given tumor T-category is a surrogate of time and that primary tumor growth and metastatic spread occur alongside and are hence correlated. Late T-category tumors are on average diagnosed in a later phase of their disease than early T-category tumors, patients are consequently more likely to be in a more advanced state of nodal progression, which in turn increases the risk of microscopic involvement of LNL—an intuition that can be quantified by the presented model. Also, the model assumes the pathways of lymphatic spread to stay the same throughout the evolution of the disease, which is probably not true for all patients, especially when presenting with very advanced tumor stages.

To the best of our knowledge, it has not been investigated how much time passes between tumor formation and diagnosis and how this varies with T-category. Although, this may initially appear as a problem, it is surprisingly not relevant—although interesting—how much time passes in the real world between two time-steps in the model. The model does not even assume that this time per time-step remains constant. It could, for example, become progressively shorter for later time-steps, accounting for the fact that a more advanced tumor also spreads faster. The time-prior’s exact shape however is harder to determine. This distribution gives, by definition, the probability to diagnose a patient after $t$ time-steps, given their T-category. However, it can be shown empirically, that support and exact shape of the time-prior have no or limited impact on the model predictions.

There have been two other studies^38,39^ from the same group that looked into modelling lymphatic metastatic progression in head and neck cancer using Markov models. The authors in those works, too, express that the length of a time-step is abstract and not necessary for modelling. The first study^38^ differs from the work presented here in that it models a LNLs state not as binary, but as a categorical variable taking on values between 0 and 4, indicating different states of involvement. With the increase of an LNL’s state, the probability of spread to the next LNL increases too. This is an interesting idea that could potentially be incorporated into our methodology as well. A shortcoming of their approach is that they assume all LNLs to have the same probabilities of evolving and metastasizing and they are not learned, but arbitrarily fixed. Also, T-category enters the model only via the number of time-steps the model is run for and the state a patient is ultimately in is modelled as observable, not hidden. The second work^39^ models T-category explicitly as a random variable and the involvement of all LNLs along a chain up to a certain LNL as binary. It is not modelled as hidden and the probabilities for progressing to the next T-category are constant, as well as the probabilities for the involvement to spread further down the chain.

The methodology presented here may be used to inform future guidelines on elective nodal CTV definition or the extent of surgical resection. However, to do so, learning of the model parameters must be based on larger training datasets of lymphatic progression patterns than the one we reconstructed from^8^. Currently, there is a lack of available training data in the form necessary for the model, which requires a table with rows of patients and columns of patient information containing T-category, whether or not each individual LNL was involved, and possibly additional risk factors that potentially have impact on nodal progression. Such data is routinely acquired in clinical practice and could be anonymized for sharing without substantial hurdles regarding patient data confidentiality. However, it is not published. Many studies only report prevalence of LNL involvement^3,6,7,26–30,40^ but omit detailed individual reports on the patterns of involvement, i.e. which LNLs were simultaneously involved. Although prevalence data can be incorporated into our model as a special case of incomplete observations ("Learning and risk assessment for incomplete diagnoses" section), it is not helpful for addressing the question how the location of macroscopic metastases impacts the risk of microscopic disease in other LNL. At the university hospital Zurich, we are currently in the process of collecting and curating such a dataset to consolidate risk predictions for ipsilateral levels I-IV and to further extend the model.

Larger data sets will allow us in the future to extend the model to include: (1) additional LNLs such as levels V and VII. This corresponds to extending the graph and thereby the set of parameters. Since these levels are more rarely involved, larger datasets for training are required. (2) other tumor locations in the head and neck region such as hypopharynx, larynx, and oral cavity. Intuitively one may expect that different primary tumor locations mainly mean different base probability rates $\tilde{b}$ while the transition probability rates $\tilde{t}$ remain similar, since they depend on lymphatic drainage between levels rather than the primary tumor location. However, only larger datasets will answer this question. Multiple tumor sites can also be incorporated into our graph-based approach with relative ease. (3) contralateral spread accounting for patient-specific observations such as midline extension of the primary tumor. Here too, one may expect the transition probability rates to remain similar between ipsilateral and contralateral side while the contralateral base probabilities are lower depending on the lateralization of the primary tumor. (4) Beyond changing the graph structure and its parameters, we would also like to include other risk factors such as HPV status, age, alcohol and nicotine abuse etc. into the model at some point in the future. (5) Apart from HNSCC, the methodology presented here may also be applied to calculate probabilities of lymphatic spread in other cancer sites such as breast or advanced stage prostate cancer.

In conclusion, we presented an interpretable probabilistic model to describe lymphatic tumor progression over time, which incorporates both the anatomy of the lymphatic drainage system as well as clinical data on lymph node involvement. It extends previous work on estimating the risk of microscopic involvement in lymph node levels by incorporating T-category as an additional risk factor. When provided with larger and more diverse datasets, the model may support clinicians in making CTV-N definition more objective and personalized.

## Supplementary Information

Supplementary Information.
