# Publisher Correction: A hidden Markov model for lymphatic tumor progression in the head and neck

**DOI:** 10.1038/s41598-021-94427-7

**Published:** 2021-07-27

**Authors:** Roman Ludwig, Bertrand Pouymayou, Panagiotis Balermpas, Jan Unkelbach

**Affiliations:** Department of Radiation Oncology, University Hospital of Zurich, Zurich, Switzerland

Correction to: *Scientific Reports* 10.1038/s41598-021-91544-1, published online 10 June 2021

The original version of this Article contained repeated errors in the Equations.

Equations 1, 4, 15, 16, 21 and 26 were incorrectly aligned.

Equation 2 and 14 contained an incorrect separator (|).

Equation 3 contained a duplication of terms.

The ${\operatorname{pa}(v)}$ in Equations 3, 4 and 5 was incorrectly given in italics.

The product ($\prod$), sum ($\sum$) and fraction operators in Equations 6, 18, 19, 20, 22, 23, 24, 27 and 31 were incorrectly given in a lower-height format.

The numbering indicating Equation 15 was omitted.

The original Equations 1, 2, 3, 4, 5, 6, 14, 15, 16, 18, 19, 20, 21, 22, 23, 24, 26, 27, 29 and 31 are included below.

1 $$\begin{aligned} P_{BN} \left( {Z_{v}^{k} = z_{v}^{k} |X_{v} = x_{v} } \right) & = \left( {z_{v}^{k} + \left( { - 1} \right)^{{z_{v}^{k} }} \cdot s_{P}^{k} } \right)\left( {1 - x_{v} } \right) \\ & \quad + \left( {( {1 - z_{v}^{k} } ) + \left( { - 1} \right)^{{1 - z_{v}^{k} }} \cdot s_{N}^{k} } \right)x_{v} \\ \end{aligned}$$

2 $$\begin{array}{*{20}c} {P_{BN} \left( {Z_{v}^{k} = 0 \, | \, X_{v} = 1} \right) = 1 - s_{N}^{k} } \\ \end{array}$$

3 $$\begin{array}{*{20}c} {P_{BN} \left( {X_{v} = x_{v} |X_{pa\left( v \right)} = x_{pa\left( v \right)} , b_{v} ,t_{pa\left( v \right)v} } \right) = x_{v} + \left( { - 1} \right)^{{x_{v} }} \left( {1 - b_{v} } \right)\left( {1 - t_{pa\left( v \right)v} } \right)^{{x_{pa\left( v \right)} }} }= x_{v} \\ + {(-1)^{x_{v}}} \left( {1 - b_{v} }\right) \left( {1 - t_{pa\left( v \right)v} } \right)^{{x_{pa\left( v \right)} }}\end{array}$$

4 $$\begin{aligned} & P_{BN} \left( {X_{v} = 0 \,| \, X_{pa\left( v \right)} = 0} \right) = 1 - b_{v} \\ & P_{BN} \left( {X_{v} = 1 \, | \, X_{pa\left( v \right)} = 0} \right) = b_{v} \\ & P_{BN} \left( {X_{v} = 0 \, | \, X_{pa\left( v \right)} = 1} \right) = \left( {1 - b_{v} } \right)\left( {1 - t_{pa\left( v \right)v} } \right) \\ & \begin{array}{*{20}c} {P_{BN} \left( {X_{v} = 1 \, | \, X_{pa\left( v \right)} = 1} \right) = 1 - \left( {1 - b_{v} } \right)\left( {1 - t_{pa\left( v \right)v} } \right)} \\ \end{array} \\ \end{aligned}$$

5 $$\begin{aligned} \begin{array}{*{20}c} {P_{BN} \left( {X_{v} = x_{v} \, |\left\{ {X_{pa\left( v \right)} = x_{pa\left( v \right)} } \right\},\left\{ {t_{pa\left( v \right)v} } \right\}, b_{v} } \right) = {x_{v} + \left( { - 1} \right)^{{x_{v} }} \left( {1 - b_{v} } \right)} \mathop \prod \limits_{p \in pa\left( v \right)} \left( {1 - t_{pv} } \right)^{{x_{p} }} } \\ \end{array} \end{aligned}$$

6 $$\begin{array}{*{20}c} {P_{BN} \left( {{\mathbf{\mathcal{Z}}} \, |\theta } \right) = \mathop \prod \limits_{n = 1}^{N} \mathop \sum \limits_{{{\varvec{x}} \in \left\{ {0,1} \right\}^{V} }} \mathop \prod \limits_{v = 1}^{V} \mathop \prod \limits_{{k \in {\mathcal{O}}}} P_{BN} \left( {z_{nv}^{k} \, | \, x_{v} } \right)P_{BN} \left( {x_{v} \, | \, \left\{ {x_{{{\text{pa}}\left( v \right)}} } \right\},\left\{ {t_{{{\text{pa}}\left( v \right)v}} } \right\}, b_{v} } \right)} \\ \end{array}$$

14 $$\begin{aligned} & P_{HMM} \left( {{\varvec{x}}\left[ {t + 1} \right] \, | \, {\varvec{x}}\left[ t \right]} \right) \\ & \quad = \mathop \prod \limits_{v \in V} Q\left( {x_{v} \left[ {t + 1} \right];x_{v} \left[ t \right]} \right)\left( {P_{BN} \left( {x_{v} \left[ {t + 1} \right]|\left\{ {x_{{{\text{pa}}\left( v \right)}} \left[ t \right]} \right\},\left\{ {\tilde{t}_{{{\text{pa}}\left( v \right)v}} } \right\}, \tilde{b}_{v} } \right)} \right)^{{1 - x_{v} \left[ t \right]}} \\ \end{aligned}$$

15 $$\begin{aligned} & Q\left( {X_{v} \left[ {t + 1} \right] = 0 \, | \,X_{v} \left[ t \right] = 0} \right) = 1 \\ & Q\left( {X_{v} \left[ {t + 1} \right] = 0 \, | \, X_{v} \left[ t \right] = 1} \right) = 0 \\ & Q\left( {X_{v} \left[ {t + 1} \right] = 1 \, | \, X_{v} \left[ t \right] = 0} \right) = 1 \\ & \begin{array}{*{20}c} {Q\left( {X_{v} \left[ {t + 1} \right] = 1 \, | \, X_{v} \left[ t \right] = 1} \right) = 1} \\ \end{array} \\ \end{aligned}$$

16 $$\begin{aligned} & P_{HMM} \left( {{\varvec{X}}\left[ {t + 1} \right] = {\varvec{\xi}}_{7} \user2{\, | \,X}\left[ t \right] = {\varvec{\xi}}_{5} } \right) \\ & \quad = Q\left( {X_{1} \left[ {t + 1} \right] = 0 \, | \, X_{1} \left[ t \right] = 0} \right)P_{BN} \left( {X_{1} \left[ {t + 1} \right] = 0 \, | \,\tilde{b}_{1} } \right)^{1} \\ & \qquad \cdot \;Q\left( {X_{2} \left[ {t + 1} \right] = 1 \, | \, X_{2} \left[ t \right] = 1} \right)P_{BN} \left( {X_{2} \left[ {t + 1} \right] = 1 \, | \, X_{1} \left[ t \right] = 0,\tilde{t}_{12} ,\tilde{b}_{2} } \right)^{0} \\ & \qquad \cdot \;Q\left( {X_{3} \left[ {t + 1} \right] = 1{\, | \,}X_{3} \left[ t \right] = 0} \right)P_{BN} \left( {X_{3} \left[ {t + 1} \right] = 1 \, | \, X_{2} \left[ t \right] = 1,\tilde{t}_{23} ,\tilde{b}_{3} } \right)^{1} \\ & \qquad \cdot \;Q\left( {X_{4} \left[ {t + 1} \right] = 0 \, | \, X_{4} \left[ t \right] = 0} \right)P_{BN} \left( {X_{4} \left[ {t + 1} \right] = 0 \, | \, X_{3} \left[ t \right] = 0,\tilde{t}_{34} ,\tilde{b}_{4} } \right)^{1} \\ & \quad = \left( {1 - \tilde{b}_{1} } \right) \cdot 1 \cdot \left( {\tilde{b}_{3} + \tilde{t}_{23} - \tilde{b}_{3} \tilde{t}_{23} } \right) \cdot \left( {1 - \tilde{b}_{4} } \right) \\ \end{aligned}$$

18 $$\begin{array}{*{20}c} {P\left( {{\varvec{z}} = {\varvec{\zeta}}_{j} } \right) = \mathop \sum \limits_{{t \in {\mathbb{T}}}} p\left( t \right) \cdot P\left( {{\varvec{z}} = {\varvec{\zeta}}_{j} ,t} \right) = \left[ {\mathop \sum \limits_{{t \in {\mathbb{T}}}} p\left( t \right) \cdot {\varvec{\pi}}^{ \top } \cdot \left( {\varvec{A}} \right)^{t} \cdot {\varvec{B}}} \right]_{j} } \\ \end{array}$$

19 $$\begin{array}{*{20}c} {P\left( {{\mathbf{\mathcal{Z}}}{|}\theta } \right) = \mathop \prod \limits_{i = 1}^{{V \cdot \left| {\mathcal{O}} \right|}} P\left( {{\varvec{\zeta}}_{i} \, | \,\theta } \right)^{{f_{i} }} } \\ \end{array}$$

20 $$\begin{array}{*{20}c} {P\left( {\theta {\,|\,}{\mathbf{\mathcal{Z}}}} \right) = \frac{{P\left( {{\mathbf{\mathcal{Z}}}{\,|\,}\theta } \right)P\left( \theta \right)}}{{\smallint P\left( {{\mathbf{\mathcal{Z}}}{\,|\,}\theta^{{\prime }} } \right)P\left( {\theta^{{\prime }} } \right)d\theta^{{\prime }} }}} \\ \end{array}$$

21 $$P\left( \theta \right) = \left\{ {\begin{array}{*{20}c} 1 & \,\,{{\text{if}}\;\theta \in {\mathcal{S}}^{{V\left( {V - 1} \right)}} } \\ 0 & {{\text{otherwise}}} \\ \end{array} } \right.$$

22 $$\begin{array}{*{20}c} {\log P\left( {{\mathbf{\mathcal{Z}}}{\,|\,}\theta } \right) = \mathop \sum \limits_{T = 1}^{4} \log \left[ {\mathop \sum \limits_{{t \in {\mathbb{T}}}} p_{T} \left( t \right) \cdot {\varvec{\pi}}^{ \top } \cdot \left( {\varvec{A}} \right)^{t} \cdot {\varvec{B}}} \right] \cdot {\varvec{f}}_{T} } \\ \end{array}$$

23 $$\begin{array}{*{20}c} {R\left( {X_{v} = 1 \, | \, {\varvec{z}},\theta } \right) = \frac{{P\left( {{\varvec{Z}} = {\varvec{z}} \, | \,X_{v} = 1,\theta } \right)P\left( {X_{v} = 1 \, | \, \theta } \right)}}{{P\left( {{\varvec{Z}} = {\varvec{z}} \, | \, \theta } \right)}} = \frac{{\mathop \sum \nolimits_{{\left\{ {i:\xi_{iv} = 1} \right\}}} P\left( {{\varvec{Z}} = {\varvec{z}} \, | \, {\varvec{\xi}}_{i} ,\theta } \right)P\left( {{\varvec{\xi}}_{i} \, | \, \theta } \right)}}{{P\left( {{\varvec{Z}} = \user2{z \, | \, }\theta } \right)}}} \\ \end{array}$$

24 $$\begin{array}{*{20}c} {{\mathbb{E}}_{{\varvec{\theta}}} \left[ {R\left( {X_{v} = 1 \, | \, {\varvec{z}}} \right)} \right] = \frac{1}{L}\mathop \sum \limits_{k = 1}^{L} R\left( {X_{v} = 1 \, | \, {\varvec{z}},\theta_{k} } \right)} \\ \end{array}$$

26 $$\begin{array}{*{20}c} {{\text{match}}\left( {{\varvec{d}},{\varvec{z}}} \right): = \left\{ {\begin{array}{*{20}c} {{\text{true}}} & {{\text{if}}\;d_{v}^{{\mathcal{O}}} = z_{v}^{{\mathcal{O}}} \vee d_{v}^{{\mathcal{O}}} = \emptyset ;\quad \forall v,O } \\ {{\text{false}}} & {{\text{else}}} \\ \end{array} } \right.} \\ \end{array}$$

27 $$\begin{array}{*{20}c} {R\left( {X_{v} = 1{\,|\,}{\varvec{d}},\theta } \right) = \frac{{P\left( {{\varvec{d}}{\,|\,}X_{v} = 1,\theta } \right)P\left( {X_{v} = 1{\,|\,}\theta } \right)}}{{P\left( {{\varvec{d}}{\,|\,}\theta } \right)}} = \frac{{\mathop \sum \nolimits_{{\left\{ {i : \xi_{iv} = 1} \right\}}} P\left( {{\varvec{d}}{\,|\,}{\varvec{\xi}}_{i} ,\theta } \right)P\left( {{\varvec{\xi}}_{i} {\,|\,}\theta } \right)}}{{P\left( {{\varvec{d}}{\,|\,}\theta } \right)}}} \\ \end{array}$$

29 $$\begin{array}{*{20}c} {P\left( {{\varvec{d}}{\,|\,}\theta } \right) = \mathop \sum \limits_{{\left\{ {j:{\text{match}}\left( 
{{\varvec{d}},{\varvec{\zeta}}_{j} } \right)} \right\}}} \left[ {\mathop \sum \limits_{{t \in {\mathbb{T}}}} p_{T} \left( t \right) \cdot {\varvec{\pi}} \cdot \left( {\varvec{A}} \right)^{t} \cdot {\varvec{B}}} \right]_{j} } \\ \end{array}$$

31 $$\begin{array}{*{20}c} {P\left( {{\varvec{d}}{\, | \,}\theta } \right) = \mathop \sum \limits_{{t \in {\mathbb{T}}}} p_{T} \left( t \right) \cdot {\varvec{\pi}} \cdot \left( {\varvec{A}} \right)^{t} \cdot {\varvec{B}} \cdot {\varvec{c}}^{{\varvec{d}}} } \\ \end{array}$$

The original Article has been corrected.

