# Supplementary material for: A hidden Markov model for lymphatic tumor progression in the head and neck
Source: Sci Rep. 2021 Jun 10;11:12261. doi: 10.1038/s41598-021-91544-1 (PMC8192955; doi:10.1038/s41598-021-91544-1)
Supplement: Supplementary file 1 — Supplementary Information. [file 41598_2021_91544_MOESM1_ESM.docx]

# Supplementary Material

## Code

The code underlying this publication here was developed by us and implemented in python. It is packaged and published on GitHub as an open-source repository under MIT license. It can be found at <https://github.com/rmnldwg/lymph>. We also provide instructions on how to install it, as well as a quick-start guide and detailed documentation [here](https://lymph-model.readthedocs.io/en/latest/index.html).

In our GitHub repository, under <https://github.com/rmnldwg/lymph/notebook>, one can also find a jupyter notebook that was used to compute all results in this paper and generate every plot shown.

## Data

The dataset reconstructed from reports about the detailed patterns of involvement from early T-category HNSCC patients with primary tumors in the oropharynx that have undergone neck dissection^8^ is given below (Table 1) in condensed form and also stored as CSV table in the aforementioned repository in a folder called data under <https://github.com/rmnldwg/lymph/notebook/data>.

| **state** | **LNL I** | **LNL II** | **LNL III** | **LNL IV** | **number of occurrences** |
| --- | --- | --- | --- | --- | --- |
| $\boldsymbol{\xi}_{1}$ | 0 | 0 | 0 | 0 | 44 |
| $\boldsymbol{\xi}_{2}$ | 0 | 0 | 0 | 1 | 1 |
| $\boldsymbol{\xi}_{3}$ | 0 | 0 | 1 | 0 | 4 |
| $\boldsymbol{\xi}_{4}$ | 0 | 0 | 1 | 1 | 1 |
| $\boldsymbol{\xi}_{5}$ | 0 | 1 | 0 | 0 | 53 |
| $\boldsymbol{\xi}_{6}$ | 0 | 1 | 0 | 1 | 3 |
| $\boldsymbol{\xi}_{7}$ | 0 | 1 | 1 | 0 | 21 |
| $\boldsymbol{\xi}_{8}$ | 0 | 1 | 1 | 1 | 11 |
| $\boldsymbol{\xi}_{9}$ | 1 | 0 | 0 | 0 | 1 |
| $\boldsymbol{\xi}_{10}$ | 1 | 0 | 0 | 1 | 2 |
| $\boldsymbol{\xi}_{11}$ | 1 | 0 | 1 | 0 | 0 |
| $\boldsymbol{\xi}_{12}$ | 1 | 0 | 1 | 1 | 0 |
| $\boldsymbol{\xi}_{13}$ | 1 | 1 | 0 | 0 | 1 |
| $\boldsymbol{\xi}_{14}$ | 1 | 1 | 0 | 1 | 0 |
| $\boldsymbol{\xi}_{15}$ | 1 | 1 | 1 | 0 | 5 |
| $\boldsymbol{\xi}_{16}$ | 1 | 1 | 1 | 1 | 0 |

Table 1: All 16 distinct states in our model of four binary random variables. Columns are the state, the involvements of LNL I to IV (0 for healthy, 1 for involved) and how often the respective state was observed.

# Appendix: Time-steps and time-prior

To add more interpretability to the time-prior $p\left( t \right)$ introduced in section 3.3, we want to give some insights here to what we think the time-steps and the distribution over them is supposed to mean.

## Interpretation of time-steps and time-priors

First, the time that passes in the real world between the abstract time-steps $t$ and $t+1$ should not be seen as a somewhat arbitrarily chosen fixed time, measured in days or weeks. To how much real-world time that corresponds for a specific patient is irrelevant for our risk assessment, although it might prove very valuable for other research on tumor growth. Also, the time between two time-steps does not need to be constant; the model makes no assumptions about this. It merely assumes the probability of transition between states to be the same from $t$ to $t+1$ and for all $t$.

The time-prior $p\left( t \right)$ is essentially the probability that a patient is diagnosed after exactly $t$ time-steps. If we knew how long a patient had cancer before getting diagnosed and we also knew how long a typical timestep for this patient and his/her type of cancer was, then we could just fix $p\left( t \right)=1$ for the appropriate number of time-steps $t$ and set $p\left( t^{'} \right)=0, \forall t^{'}\neq t$. Since it is likely almost never known, we need to spread the probability over a range of time-steps, reflecting the fact that the diagnose of cancer happens spontaneously, e.g. during a routine checkup.

## Impact of shape and length of the time-prior

It turns out that length and shape of $p\left( t \right)$ have almost no effect on the risk predictions as long as we are not concerned with different T-categories. So, if we learn our parameters from a dataset that only contains T1 patients and then compute risks for T1 patients only, the result will not differ almost regardless of the time-prior that was used for learning and risk assessment. Only too few time-steps may pose a problem, since then the system might not be able to spread to all LNLs via all pathways. And too many time-steps could introduce numerical problems, because the learned probability rates $\tilde{b}_{v}$ and $\tilde{t}_{\mathrm{pa} \left( v \right)v}$ become smaller for longer time-priors.


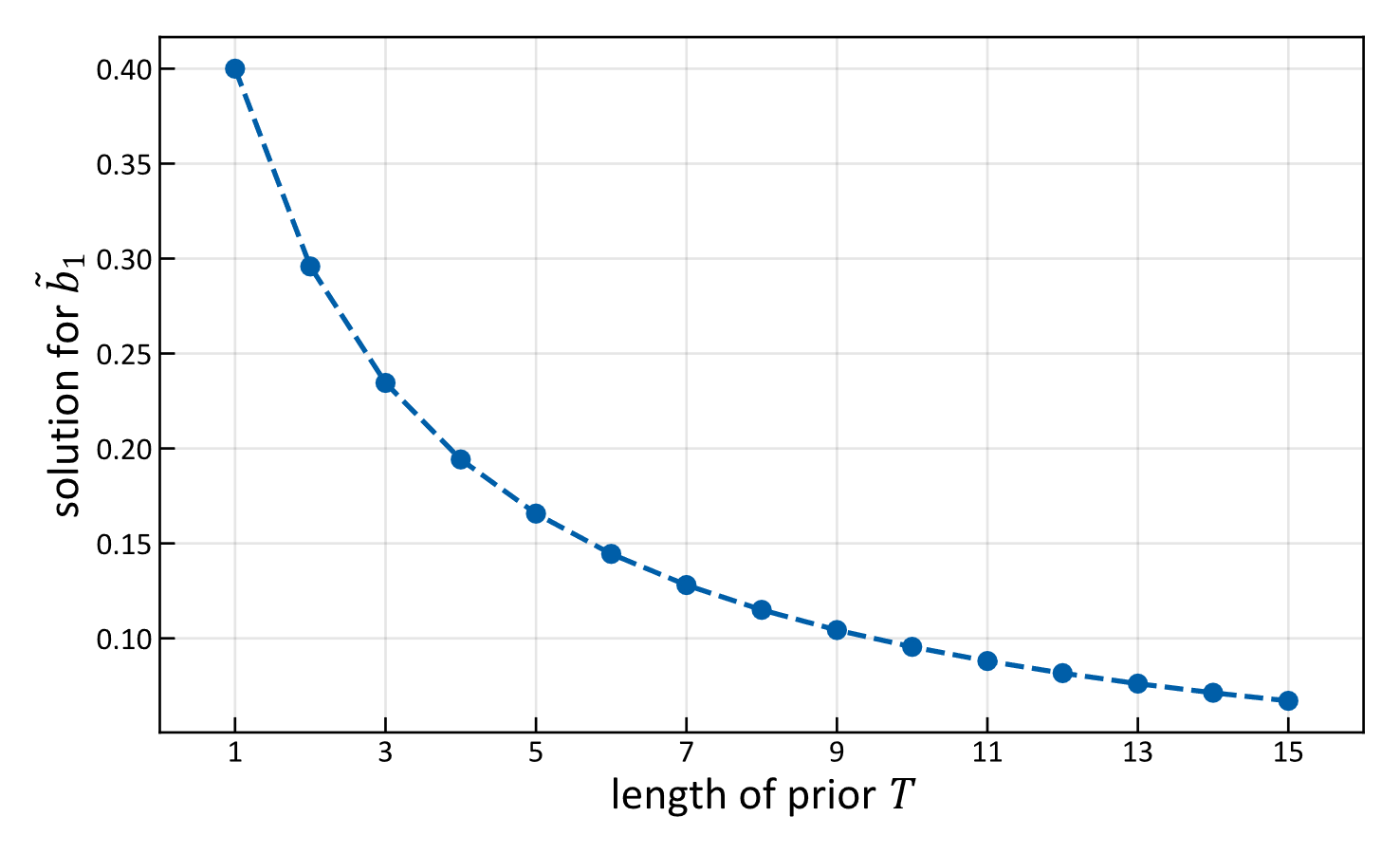


Figure A1: Solutions to equation$\left( A3 \right)$ for the base probability rate $\tilde{b}_{1}$ given a $p^{\star}$ of 0.4 and $T$ increasing from 1 to 15.

To understand the impact of the number of time-steps $T$ on the results, we looked at a simple analytical model: Assume that there is a system with only one LNL that the primary tumor can spread to that is empirically involved with probability $p^{*}=0.4$. For this situation, we can now derive how the base probability rate $\tilde{b}_{1}$ changes for a uniform time-prior

$$\begin{aligned} p\left( t \right)=\frac{1}{T}\quad\text{for} t\in\left\{ 1, 2, \ldots, T \right\}\#\left( A1 \right) \end{aligned}$$

if we vary the total number of time-steps $T$. We can write $p^{*}$ as

$$\begin{aligned} p^{\star}&=\frac{1}{T}\sum_{t=1}^{T} \left( \begin{matrix} 1 & 0 \end{matrix} \right)\cdot\left[ \begin{matrix} \left( 1-\tilde{b}_{1} \right) & \tilde{b}_{1} \\ 0 & 1 \end{matrix} \right]^{t}\cdot\left( \begin{matrix} 0 \\ 1 \end{matrix} \right) \\ &=\frac{1}{T}\sum_{t=1}^{T} \left( \begin{matrix} 1 & 0 \end{matrix} \right)\cdot\left[ \begin{matrix} \left( 1-\tilde{b}_{1} \right)^{t} & 1-\left( 1-\tilde{b}_{1} \right)^{t} \\ 0 & 1 \end{matrix} \right]\cdot\left( \begin{matrix} 0 \\ 1 \end{matrix} \right) \\ &=\frac{1}{T}\sum_{t=1}^{T} \left[ 1-\left( 1-\tilde{b}_{1} \right)^{t} \right]=1-\frac{1}{T}\sum_{t=1}^{T} \left( 1-\tilde{b}_{1} \right)^{t}\#\left( A2 \right) \end{aligned}$$

The right-hand side essentially contains the partial sum of the geometric series and can easily be computed to yield

$$\begin{aligned} p^{\star}=1-\frac{\left( 1-\tilde{b}_{1} \right)\left( 1-\left( 1-\tilde{b}_{1} \right)^{T} \right)}{\tilde{b}_{1}T}\#\left( A3 \right) \end{aligned}$$

It is not possible to analytically solve for $\tilde{b}_{1}$ in the case of arbitrary $T$, but numerical solutions are very easy to find and are plotted in Figure A1. This confirms the intuition, that the base and transition probability rates become smaller when the total time over which the tumor spreads is divided into more but shorter time-steps.

Now we compare this idealized result to the decay of the probability rates for the full system. To that end, the model with LNLs I-IV was trained as in the same way as for Figures 5-8, but with differently long uniform time-priors instead of a Binomial prior. So, the probability for every time-step is $p\left( t \right)=1/T$ for all $t\geq1$, but zero for the starting state $\boldsymbol{\pi}$. Figure A2 shows the expected value of the parameters as a function of $T$. It is important to stress again that the risk predicted by the models using all those different-length uniform time-priors was the same for $T\geq2$. For the one-step model with $T=1$ the risk prediction of a LNL does not depend on the diagnose. For example, we expect the risk in level III is higher, when level II is involved, due to the spread from LNL II to III. With such a short time-prior, however, the model cannot capture this, and all risk predictions will just yield the prevalence of involvement. This effect can be seen in Figure A3 and shows what has been stated earlier: The support of the time-prior has little effect on the model’s predictions, as long as it is sufficient to capture the spread through the lymphatic system.


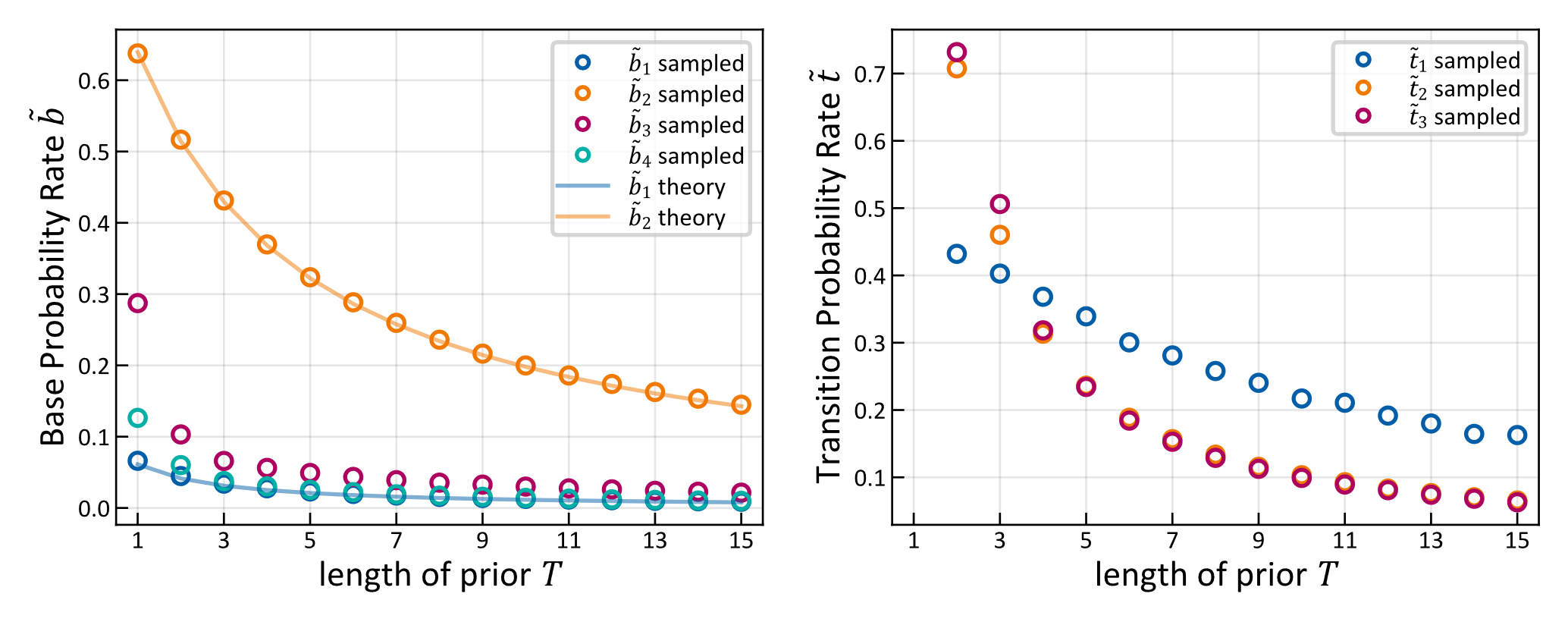


Figure A2: Decay of base probability rates as a function of the number of time-steps the time-prior has. Circles depict the results from learning the same dataset with different time-priors while solid lines show the analytical result starting with a $p^{\star}$ corresponding to the prevalence of involvement of LNL I and II respectively.

The theoretical result in equation $\left( A3 \right)$ is applicable to the parameter $\tilde{b}_{1}$, and approximately to $\tilde{b}_{2}$ since involvement of level II is driven by direct infiltration from the primary tumor rather than transition from level I. For levels III and IV, the theory is not applicable as they have two relevant parent nodes. The solid lines in Figure A2 show agreement of the theoretical result with the sampling based training of the full model (circles), where the probabilities $p^{\star}$ were set to 6.1% and 63.9%, corresponding to the prevalence of level I and II involvement in the dataset, respectively.

This again shows that, while looking at one T-category only, the time-prior’s parameters overdetermine the system. For any choice of $T$, the base and transition probability rates can be adjusted such that the Hidden Markov Model is equivalent to the Bayesian network’s performance. Only if we want to distinguish between patients of different T-category the HMM can outperform the BN.


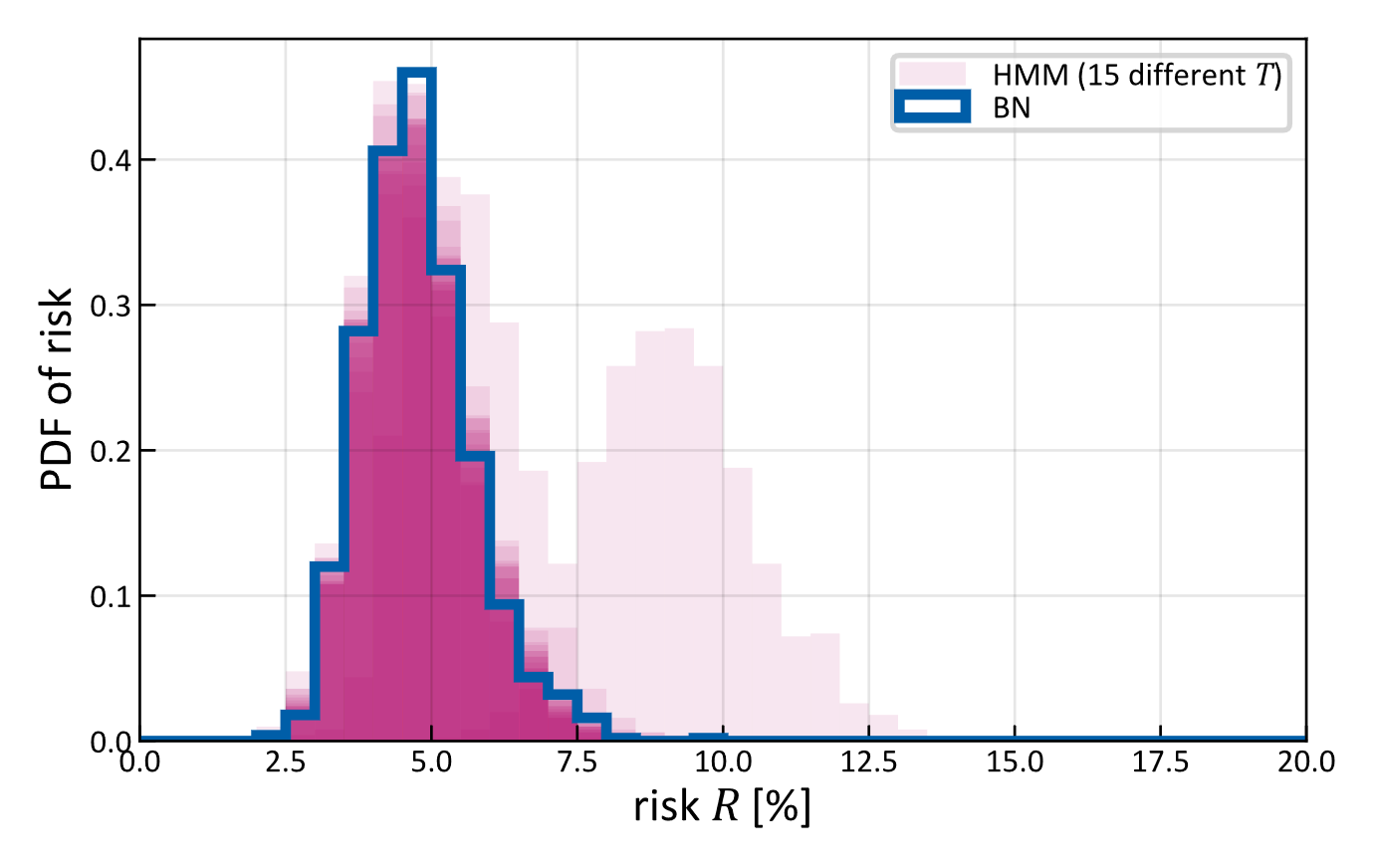


Figure A3: Prediction for the risk of involvement in LNL III, given that no other LNL was observed to be involved. Computed by training our hidden Markov model with 15 different-length uniform time-priors (transparent red) as well as the Bayesian network model (blue line). The one outlier is the HMM with a time-prior covering only one time-step. It is centered on the prevalence of involvement for LNL III, regardless of the given diagnose.
